# Development and visualization of a risk prediction model for metabolic syndrome: a longitudinal cohort study based on health check-up data in China

**DOI:** 10.3389/fnut.2023.1286654

**Published:** 2023-11-21

**Authors:** Wenxi Liu, Xiao Tang, Tongcheng Cui, Hui Zhao, Guirong Song

**Affiliations:** ^1^Department of Health Statistics, School of Public Health, Dalian Medical University, Dalian, China; ^2^Department of Health Examination Center, The Second Affiliated Hospital of Dalian Medical University, Dalian, China

**Keywords:** metabolic syndrome, the cox proportional hazards regression, random survival forest, risk prediction model, longitudinal data

## Abstract

**Aim:**

Our study aimed to construct a practical risk prediction model for metabolic syndrome (MetS) based on the longitudinal health check-up data, considering both the baseline level of physical examination indicators and their annual average cumulative exposure, and to provide some theoretical basis for the health management of Mets.

**Methods:**

The prediction model was constructed in male and female cohorts, separately. The shared set of predictive variables screened out from 49 important physical examination indicators by the univariate Cox model, Lasso-Cox model and the RSF algorithm collectively was further screened by Cox stepwise regression method. The screened predictors were used to construct prediction model by the Cox proportional hazards regression model and RSF model, respectively. Subsequently, the better method would be selected to develop final MetS predictive model according to comprehensive comparison and evaluation. Finally, the optimal model was validated internally and externally by the time-dependent ROC curve (tdROC) and concordance indexes (C-indexes). The constructed predictive model was converted to a web-based prediction calculator using the “shiny” package of the R4.2.1 software.

**Results:**

A total of 15 predictors were screened in the male cohort and 9 predictors in the female cohort. In both male and female cohorts, the prediction error curve of the RSF model was consistently lower than that of the Cox proportional hazards regression model, and the integrated Brier score (IBS) of the RSF model was smaller, therefore, the RSF model was used to develop the final prediction model. Internal validation of the RSF model showed that the area under the curve (AUC) of tdROC for 1 year, 3 years and 5 years in the male cohort were 0.979, 0.991, and 0.983, and AUCs in the female cohort were 0.959, 0.975, and 0.978, respectively, the C-indexes calculated by 500 bootstraps of the male and female cohort RSF models are above 0.7. The external validation also showed that the model has good predictive ability.

**Conclusion:**

The risk predictive model for MetS constructed by RSF in this study is more stable and reliable than Cox proportional hazards regression model, and the model based on multiple screening of routine physical examination indicators has performed well in both internal and external data, and has certain clinical application value.

## 1 Introduction

Metabolic Syndrome (MetS) is a group of clinical syndromes that includes obesity, hyperglycemia, dyslipidemia and hypertension, which seriously affect the health of the body. It is a combination of interrelated metabolic risk factors that directly promote the occurrence of atherosclerotic cardiovascular disease and increase the risk of type 2 diabetes (1). Studies have reported that compared to those without MetS, individuals with MetS have a 1.26-fold increased risk of all-cause mortality, a 1.41-fold increased risk of cardiovascular disease, and a 5-fold increased risk of type2 diabetes. And MetS has become an important risk factor for a variety of chronic noncommunicable diseases (2, 3). The incidence of MetS is on the rise worldwide, with an adult prevalence of approximately 25%, and MetS has become a serious public health issue of global concern (4). A considerable proportion of Chinese adults also suffer from MetS (5), and a meta-analysis of 28 Chinese studies on the epidemiology of MetS published between 2014 and 2017 showed that the prevalence of MetS was 21.90% (6). Furthermore, the pathogenesis of MetS is complex, and patients in the early stage generally have no obvious clinical symptoms, making it difficult to detect and easy to overlook (7). Therefore, gaining an in-depth understanding of the risk factors for the occurrence of MetS, early assessment and prediction of the risk of MetS, and timely identification of high-risk individuals for MetS are conducive to early and accurate intervention, and reducing the occurrence of MetS-related chronic diseases.

MetS is a disorder characterized by the aggregation of multiple risk factors (8), and its onset is affected by a variety of factors, so it is of great help to screen out important predictors in many related indicators, and construct of MetS risk prediction models for the prevention and treatment of MetS. Data from health checkup can provide some important physical examination indicators related to MetS. At present, studies at home and abroad have found a strong correlation between physical examination indicators such as blood lipids, blood sugar, blood uric acid, and hemoglobin and MetS. A retrospective cohort study based on health examination data also constructed a MetS risk prediction model using five physical examination indicators including body mass index, age, total cholesterol, alanine aminotransferase, and serum uric acid (9). However, most of these studies were based on cross-sectional data from one health examination, or the baseline level of physical examination indicators in cohort data as a predictor to construct MetS risk prediction models, only using a single measurement for each predictor (10). In fact, due to factors such as aging, the use of medications or other medical methods, or lifestyle changes, an individual’s physical examination indicators may not always be maintained at the baseline level, but will change over time. Existing studies have largely ignored the impact of dynamic changes in these physical examination indicators on the development of MetS, thereby a MetS prediction model established only based on the baseline level of health examination indicators, which does not conform to the actual situation, cannot accurately evaluate the impact of health examination indicators on the future occurrence of MetS and is difficult to accurately predict the incidence risk of MetS. This approach has limitations and deficiencies, and is difficult to provide a clear warning for the early prevention of MetS and lacks practicality.

Currently, there are few studies on MetS health management risk prediction related to MetS. Methodologically, the logistic regression model was applied to build the MetS risk prediction model in the study of Yang et al. (11) based on a Taiwanese health examination population in China, which could not consider the truncation of survival data. Although the Cox proportional hazards regression model was used in the study of Zhang (12) at Shandong University, which could analyze the data with truncated values and consider the effects of multiple factors on survival rate, the Cox proportional hazards regression requires the data to meet the assumption of proportional hazards (13). When the data do not comply with the prerequisites, analysis needs to be performed by making the data meet its assumptions through stratification or data transformation, which actually greatly limits its applicability, therefore, this modeling method may not be applicable to all health examination data.

With the rise of medical big data and the continuous development of information technology, exploring predictive factors is gradually revealing its value from the data perspective (14, 15). Machine learning (ML), as an emerging multi-field interdisciplinary discipline, involving statistics, probability theory, algorithm complexity theory and so on, has played a significant role in the various aspects of medical field (16). Random Forest (RF) is a relatively new machine learning model (a nonlinear tree-based model). In 2008, Ishwaran et al. (17) combined the RF method with traditional survival analysis to construct the Random Survival Forest (RSF) model (18), which can overcome the weakness of traditional survival analysis methods and has a wider range of applications. RSF is a data-driven learning algorithm that is completely nonparametric and can objectively evaluate nonlinear effects and interactions between variables. RSF can also be used for variable selection, ranking the importance of variables by variable importance (VIMP) or minimal depth, to identify risk factors. It has shown advantages over traditional models in several studies (19–22). Therefore, our study constructed MetS risk prediction models based on RSF and Cox proportional hazards regression, and selected the optimal modeling method.

This study made full use of the longitudinal health checkup data of the healthy physical examination population, considering both the baseline level of physical examination indicators and their annual average cumulative exposure, and established MetS risk prediction models based on both RSF and traditional Cox proportional hazards regression. The two models were compared and evaluated comprehensively, and the optimal MetS risk prediction model was finally selected to develop a risk prediction tool for MetS health management. This tool aims to identify and screen high-risk individuals with MetS early and accurately and effectively manage their health to reduce the burden of chronic diseases. The method can also be popularized and applied to other chronic disease risk prediction model studies, providing scientific basis for the prevention of a chronic diseases.

## 2 Materials and methods

### 2.1 Study design and data source

This was a retrospective longitudinal cohort study and the data on health check-ups from January 2011 to December 2021 at the health check-ups centre of the Second Hospital Affiliated with Dalian Medical University in Dalian were obtained, which contains various basic information and physical examination indicators of health examiners.

The initial health check-up data for each subject were defined as baseline data. A total of 5,691 subjects were initially included based on the following criteria: (1) the baseline age ranged from 20 to 60 years; (2) no history of cardiovascular and cerebrovascular diseases, diabetes, viral hepatitis, liver cirrhosis, autoimmune liver disease, renal disease or rheumatic disease at baseline; (3) no a diagnosis of MetS at baseline; and (4) no missing baseline measurements related to the study: including metabolic components and other blood biochemical indicators. Next, the following criteria were used to exclude subjects: (1) those who were not followed up and (2) those who had missing data related to MetS components, or other blood biochemical indicators or who were diagnosed with any of the above-mentioned diseases during follow-up. Finally 5,455 subjects were admitted to the valid analysis cohort used to create predictive model, including 1,527 males and 3,928 females (Supplementary Figure S1). Follow-up would be terminated if MetS occurred during the follow-up period. The study ended at the end of December 2021.

The external validation cohort was made up of the health examination data from Dalian Central Hospital, its inclusion and exclusion criteria are the same as internal validation data.

### 2.2 Measurements

The health check-ups data used in this study included routine physical examination, including sex, age, height, weight, systolic blood pressure, diastolic blood pressure, family history and medical history, and biochemical measures, such as fasting plasma glucose (FPG), triglycerides (TG), high-density lipoprotein cholesterol (HDL-C), aspartate transferase (AST), and so on. All these measuring methods have been formally described elsewhere (23).

### 2.3 Outcomes

The outcome of interest in this study was the occurrence of MetS. The 2009 Joint Interim Statement criteria were used to diagnose MetS. Subjects who had three or more of the following components were diagnosed with MetS: (1) Obesity: BMI ≥ 25 kg/m2. As waist circumference (WC) measurements were not obtained during the health check-up, BMI was utilized as a substitute to assess obesity in our study; (2) Elevated blood pressure, SBP ≥ 135 mmHg or DBP ≥ 85 mmHg, or drug treatment; (3) TG ≥ 1.7 mmol/L or drug treatment; (4) HDL-C < 1.0 mmol/L (male) and HDL-C < 1.30 mmol/L (female) or drug treatment for reduced HDL-C; and (5) FPG ≥ 5.6 mmol/L or drug treatment.

### 2.4 Predictor variables

In order to minimize the model bias caused by misidentification or omission of important predictor variables, as many potential predictor variables as possible are generally included and then screened. In this study, through extensive review of relevant literature and considering the actual situations, 25 physical examination indicators that may be associated with MetS were summarized. Except age, the annual average cumulative exposures of other physical examination indicators were calculated separately. Finally, a total of 49 important physical examination indicators needed to be considered in this study (Supplementary Table S1).

Using the area under the curve (AUC) to estimate the cumulative exposure of important physical examination indicators, the average exposure of the physical examination indicators of two consecutive physical examinations was multiplied by interval of physical examination time to obtain the area under the curve. The AUCs of all two adjacent physical examination indicators of the individual during the follow-up period were accumulated and divided by the individual’s follow-up years to obtain the annual average cumulative exposure, as shown in Supplementary Figure S2.

### 2.5 Statistical analyses

SAS 9.4 was used to organize the health examination data and establish a longitudinal analysis database. Data were analyzed using R software (version 4.2.1). Mean and standard deviation were used to describe continuous variables that followed a normal distribution, and median and interquartile range [M(P_25_, P_75_)] were used to describe continuous variables that do not follow a normal distribution. Frequency or percentage was used to describe categorical variables. Differences in characteristics between MetS groups and non-MetS groups were tested using the Wilcoxon rank test, or the chi-square test.

Due to the substantial difference in sample size between males and females in this study, as well as sex differences in levels of many predictor variables, separate cohorts were established for males and females, and the target variables were screened and their respective predictive models were constructed.

#### 2.5.1 Screening predictor variables

In separate male and female cohorts, the univariate Cox model, Lasso-Cox model, and the RSF algorithm were all used to preliminarily screen 49 important physical examination indicators included in the study and then a shared set of target variables was selected based on the results of above three methods.

The function of cv.glmnet in the “glmnet” package of R4.2.1 software was used to perform the Lasso-Cox regression model and obtain the mean square error (MSE) of each variable under different penalty parameter λ, and the optimal variable selection results were obtained under the λ corresponding to the minimum MSE.

The “randomForestSRC” package of R4.2.1 software was used to perform RSF, and select the number of survival trees, set different ntrees, run the RSF model respectively, and conduct several simulation iterations to obtain the error rate of the RSF model, respectively. The lower the error rate, the better the model fit. Considering the stability of RSF model and the cost of running time, the parameter of ntree was set as 1,000 initially, then optimal parameter of ntree was defined according to the minimum error rate. The RSF model was constructed under the optimal parameter of ntree, and the VIMP of each target variables was calculated. With the forward variable selection method, 49 target variables were introduced one by one according to the VIMP of variables, and the error rate under different combinations of target variables could be obtained. The optimal variable selection results were obtained based on the minimum error rate.

Subsequently, the shared set of target variables was further screened by Cox stepwise regression method (forward, backward, and forward-backward mixed method) and optimal subset regression method. Finally, the Akaike Information Criterion (AIC) of each model was compared to determine the final target variables. The smaller the AIC criterion, the better the model fit.

#### 2.5.2 Comparison of cox proportional hazards regression model with RSF model

The Cox proportional hazards regression model and RSF model, were constructed with the predictors screened out as independent variables, and whether MetS occurred (1 for yes, 0 for no) and the corresponding follow-up time (in years) as dependent variables. The Schoenfeld residual method was used to test the proportional hazard (PH) assumption of the Cox proportional hazards regression model, and the prediction error curves of the twomodels were plotted using the “pec” package of R4.2.1 software. The integrated Brier score (IBS) can evaluate the accuracy of models. and the smaller the score, the higher the predictive accuracy of the model. The IBS in this study was calculated based on the method of 500 bootstraps. According to the above evaluation indicators, the better method would be selected to develop final MetS predictive model.

#### 2.5.3 Validation of model

The time-dependent ROC curve (tdROC) and concordance indexes (C-indexes) were used to evaluate the discrimination ability of the model. Generally, the larger the area under the curve (AUC), the better the model’s discrimination ability. This study used the “timeROC” package of R4.2.1 to draw the tdROC curve of the model in the internal data at 1, 3, and 5 years and calculate the AUC at each time point. The C-indexes can also be used to evaluate the discrimination ability of the model. This study evaluates the predictive ability of the model based on the C-indexes calculated from 500 bootstraps.

In order to evaluate the generalization ability of the model, that is the adaptability of the model to data from other source, this study performed external validation of the constructed model using physical examination data from the health check-ups center of another tertiary hospital in Dalian. Similarly, the area under the tdROC curve was used to evaluate the discrimination ability of the model based on the external data.

#### 2.5.4 Making MetS risk prediction tool

The constructed predictive model was converted to a web-based prediction calculator using the “shiny” package of the R4.2.1 software. Clinicians can access this prediction tool on the http://. They only need to input the individual’s relevant physical examination indicators, the server can output the prediction results, that is, the individual’s risk of disease over time. Simplifying the complex model and improving the utilization of the predictive models can help clinicians make decisions.

## 3 Results

### 3.1 Basic characteristics

This study analyzed a cohort of 5,455 subjects, with a median age of 33 (24, 25) years at baseline and a median follow-up duration of 2.33 (1.04, 4.95) years. During the follow-up period, 1786 individuals developed MetS, resulting in a cumulative incidence of 32.74% and an incidence density of 10.09 per 100 person-years. The male cohort included 1,527 individuals (27.99%) with a median age of 38 (26, 27) years at baseline and a median follow-up duration of 1.39 (0.91. 4.01) years. Of these, 671 individuals developed MetS, resulting in a cumulative incidence of 43.94% and an incidence density of 16.71 per 100 person-years. The female cohort included 3,928 individuals (72.01%) with a median age of 32 (28, 29) years at baseline and a median follow-up duration of 3.01 (1.12, 5.03) years. Of these, 1,115 individuals developed with MetS, resulting in a cumulative incidence of 28.39% and an incidence density of 8.15 per 100 person-years.

In the male cohort, except for TBIL, ALB, CR, HB, RBC, PLT, UREA, ann_CumALB, ann Cum_CR, ann_CumPLT, ann_CumTP and ann_CumUREA, there were statistically significant differences in other potential pridictor variables between Mets group and non-Mets group (*p* < 0.05). In the female cohort, except for ALB, CR, UREA, ann_CumALB, ann_CumCR and ann _CumUREA, there were statistically significant differences in other potential pridictor variables between MetS group and non-MetS group (*p* < 0.05). The results are shown in Supplementary Tables S2 and S3.

### 3.2 Screening predictor variables

#### 3.2.1 Preliminarily screening

A total of 39 variables were preliminarily screened out from the male cohort and 41 variables were preliminarily screened out from the female cohort by the univariate Cox model with significant level less than 0.05 as shown in Supplementary Tables S4 and S5.

A total of 34 variables were preliminarily screened out from the male cohort and 39 variables were preliminarily screened out from the female cohort by the Lasso-Cox regression model under the penalty parameter (λ) corresponding to the minimum MSE. Some results of MetS prediction variables screened by Lasso-Cox Model are shown in Supplementary Figure S3.

In male cohort, the error rate of RSF, about 21.31%, reached the lowest, when ntree = 800. In female cohort, the lowest error rate was 14.89% at ntree = 700. A RSF model was constructed according to the optimal parameter ntree = 800 in the male cohort, and the VIMP of each target variable was calculated. The results showed that ann_CumFPG ranked the first in importance, following with ann_CumTG, ann_CumHDLC, ann_CumSBP, ann_CumDBP, and the least important target variable was CR. A RSF model was constructed according to the optimal parameter ntree = 700 in the female cohort, and the VIMP of each target variable was calculated. The results showed that ann_CumHDLC ranked the first in importance, following with ann _CumFPG, ann_CumTG, ann_CumSBP, ann_CumDBP, and the least important target variable was ALT. When the RSF model contained 22 for males and 14 target variables for females respectively, the error rates, 20.64 and 14.18%, were smallest. Therefore, the top 22 target variables were preliminarily screened out from the male cohort and the top 14 target variables were preliminarily screened out from the female cohort by the RSF model, as shown in Supplementary Tables S6 and S7.

In summary, a total of 19 shared target variables selected by the above three methods in male cohort, as well as 12 shared target variables in female cohort. Details are shown in Supplementary Tables S8 and S9.

#### 3.2.2 Secondary screening

Considering both the AIC and the number of variables included in the model, a total of 15 variables were screened out from the male cohort by secondary screening, and nine variables from the female cohort. Details are shown in Tables 1 and 2.

**Table 1:** Summary of secondary screening of MetS predictors for the male cohort.

Variable selection method	Predictors	Number of variables	AIC value
Forward	AGE, BMI, SBP, DBP, FPG, TG, HDLC, CH, TP, ann_CumSBP, ann_CumDBP, ann_CumFPG, ann_CumTG, ann_CumHDLC, ann_CumLDLC, ann_CumAST, ann_CumGGT, ann_CumCH, ann_CumRBC	19	8121.67
Backward	AGE, DBP, TG, HDLC, TP, ann_CumSBP, ann_CumDBP, ann_CumFPG, ann_CumTG, ann_CumHDLC, ann_CumLDLC, ann_CumAST, ann_CumGGT, ann_CumCH, ann_CumRBC	15	8115.78
Forward backward mixed	AGE, DBP, TG, HDLC, TP, ann_CumSBP, ann_CumDBP, ann_CumFPG, ann_CumTG, ann_CumHDLC, ann_CumLDLC, ann_CumAST, ann_CumGGT, ann_CumCH, ann_CumRBC	15	8115.78
Optimal subset regression	AGE, DBP, TG, HDLC, TP, ann_CumSBP, ann_CumDBP, ann_CumFPG, ann_CumTG, ann_CumHDLC, ann_CumLDLC, ann_CumAST, ann_CumGGT, ann_CumCH, ann_CumRBC	15	8115.783

**Table 2:** Summary of secondary screening of MetS predictors for the female cohort.

Variable selection method	Predictors	Number of variables	AIC value
Forward	AGE, SBP, FPG, HDLC, ann_CumSBP, ann_CumDBP, ann_CumFPG, ann_CumTG, ann_CumHDLC, ann_CumLDLC, ann_CumCH, ann_CumTP	12	15122.9
Backward	AGE, SBP, HDLC, ann_CumSBP, ann_CumDBP, ann_CumFPG, ann_CumTG, ann_CumHDLC, ann_CumLDLC, ann_CumCH, ann_CumTP	11	15121.04
Forward backward mixed	AGE, SBP, HDLC, ann_CumSBP, ann_CumDBP, ann_CumFPG, ann_CumTG, ann_CumHDLC, ann_CumLDLC, ann_CumCH, ann_CumTP	11	15121.04
Optimal subset regression	AGE, HDLC, ann_CumSBP, ann_CumFPG, ann_CumTG, ann_CumHDLC, ann_CumLDLC, ann_CumCH, ann_CumTP	9	15126.55

Furthermore, the variance inflation factor (VIF) of each predictor variable was less than 10 after collinearity diagnosis, indicating no strong collinearity among these variables.

### 3.3 Building MetS risk prediction model

#### 3.3.1 Comparison of cox proportional hazards regression model with RSF model

**Figure 1:**
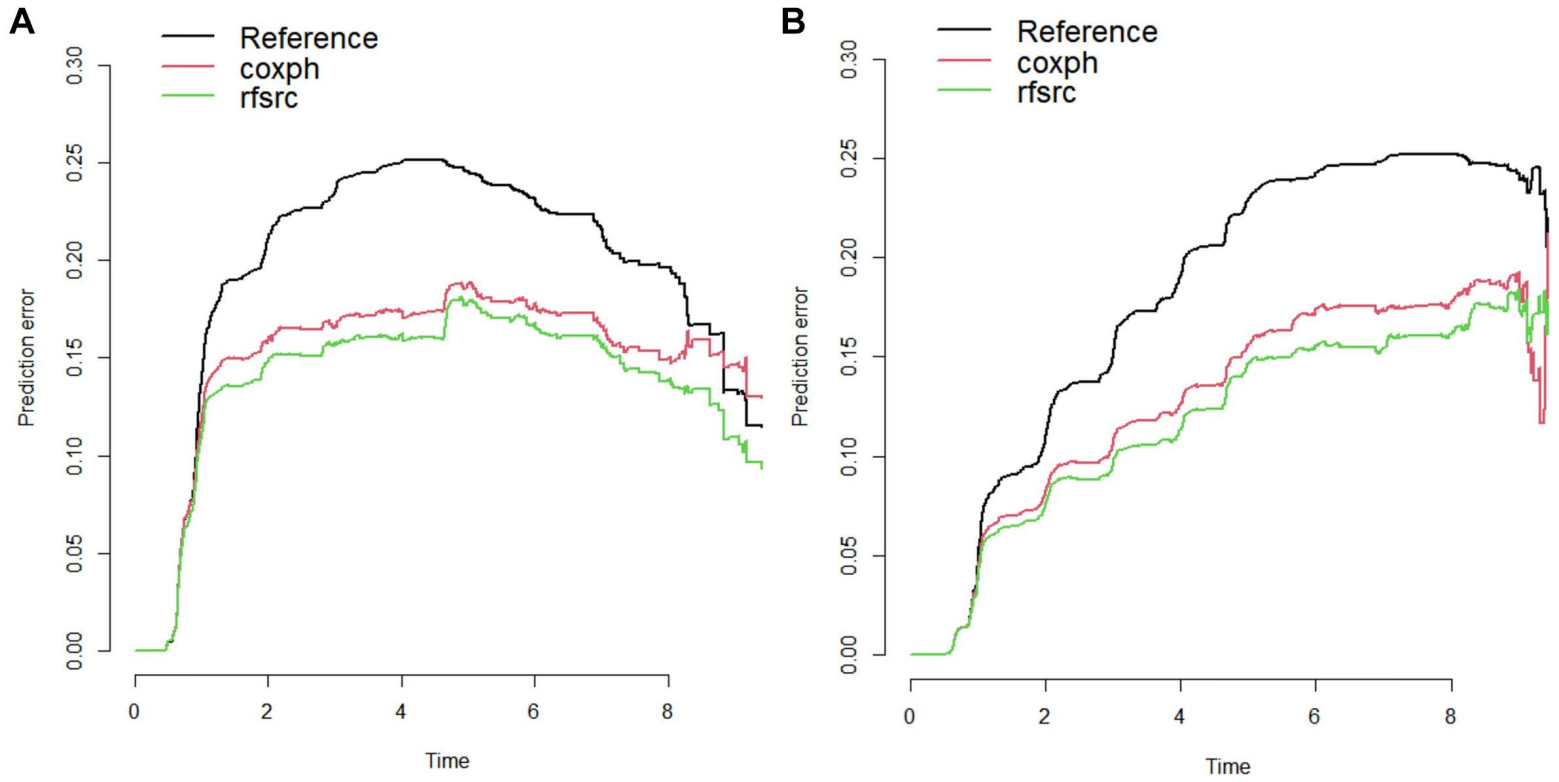
Prediction error curve of Cox proportional hazards regression model and RSF model in the male **(A)** and female **(B)** cohorts.

The Schoenfeld residual test showed that some independent variables in the Cox proportional hazards regression models in both male and female cohorts had residuals related to time (*p* < 0.1) (Supplementary Figure S4), so some independent variables did not meet the PH assumption. The prediction error curves of models in both the male and female cohort showed that the prediction error curve of the RSF model was consistently lower than that of the Cox proportional hazards regression model, and IBS of RSF models in both the male and female cohort were smaller as shown in Figure 1 and Table 3. The above results together indicate that the predictive model constructed by RSF method in this study is more stable and reliable than Cox proportional hazards regression model.

**Table 3:** The IBS of Cox proportional hazards regression model and RSF model.

The integrated brier score	Cox proportional hazards regression model	The RSF model
Male cohort	0.150	0.137
Female cohort	0.127	0.117

#### 3.3.2 Mets prediction model development by RSF

A total of 15 variables screened out from the male cohort and 9 variables from the female cohort by secondary screening were used as predictors to construct final MetS prediction model by RSF.

**Figure 2:**
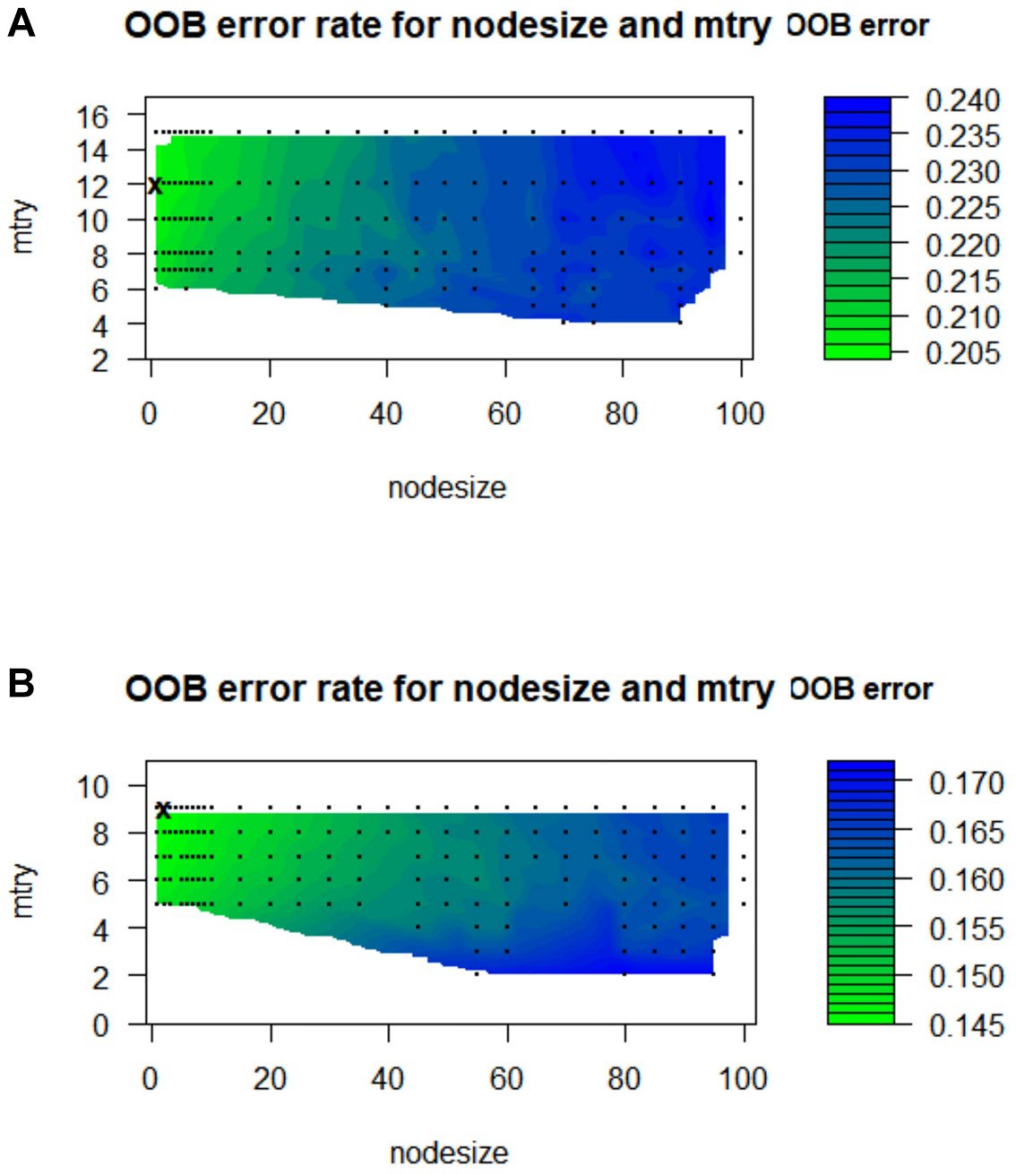
Tuning parameter diagram of RSF model in the male **(A)** and female **(B)** cohorts.

Considering that a large value of ntree may increase time costs and overfitting risks, the subsequent model was based on ntree =1,000. In addition, in the male cohort, through grid search, it was found that the minimum out-of-bag error rate of the model reached 20.54%, when the mtry value is 12 and the nodesize value is 1 (Figure 2), and we constructed the RSF model with these parameters in the male cohort. Similarly, in the female cohort, through grid search, it was found that the minimum out-of-bag error rate of the model reached 14.51% when the mtry value is 9 and the nodesize value is 3 (Figure 2), and the RSF model in the female cohort was constructed with these parameters. The specific construction parameters of RSF model in the male and female cohort are shown in Supplementary Tables S10 and S11.

**Figure 3:**
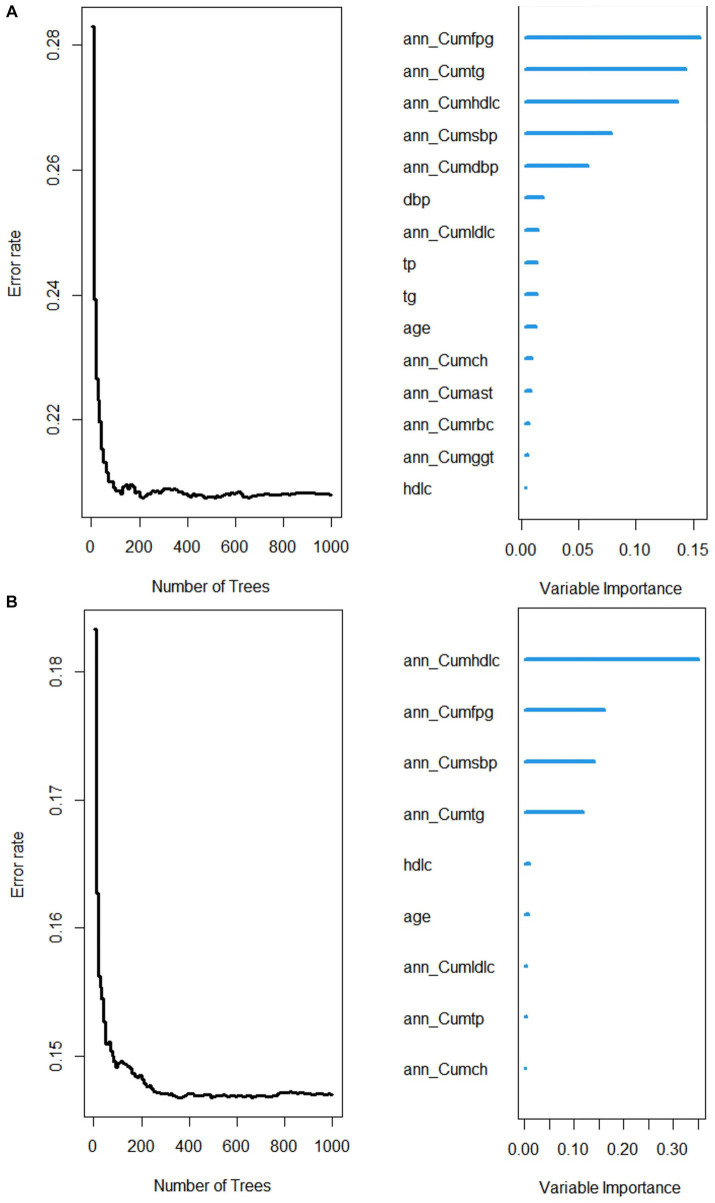
Scoring plot of variables importance for the male **(A)** and female **(B)** cohort RSF models.

As shown in Figure 3, a variable with a higher score of importance showed a greater effect on the incidence of MetS. In the male cohort model, the importance of each variable affecting the incidence of MetS was ranked from high to low as ann_CumFPG, ann_CumTG, ann_CumHDLC, ann_CumSBP, ann_CumDBP, DBP, ann CumLDLC, TP, TG, AGE, ann_CumCH, ann_CumAST, ann_CumRBC, ann_ CumGGT, HDLC. In the female cohort model, the importance of each variable affecting the incidence of Mets was ranked from high to low as ann_CumHDLC, ann _CumFPG, ann_CumSBP, ann_CumTG, HDLC, AGE, ann_CumLDLC, ann_CumTP, ann _CumCH.

### 3.4 Validation of the RSF model

#### 3.4.1 Internal validation

**Figure 4:**
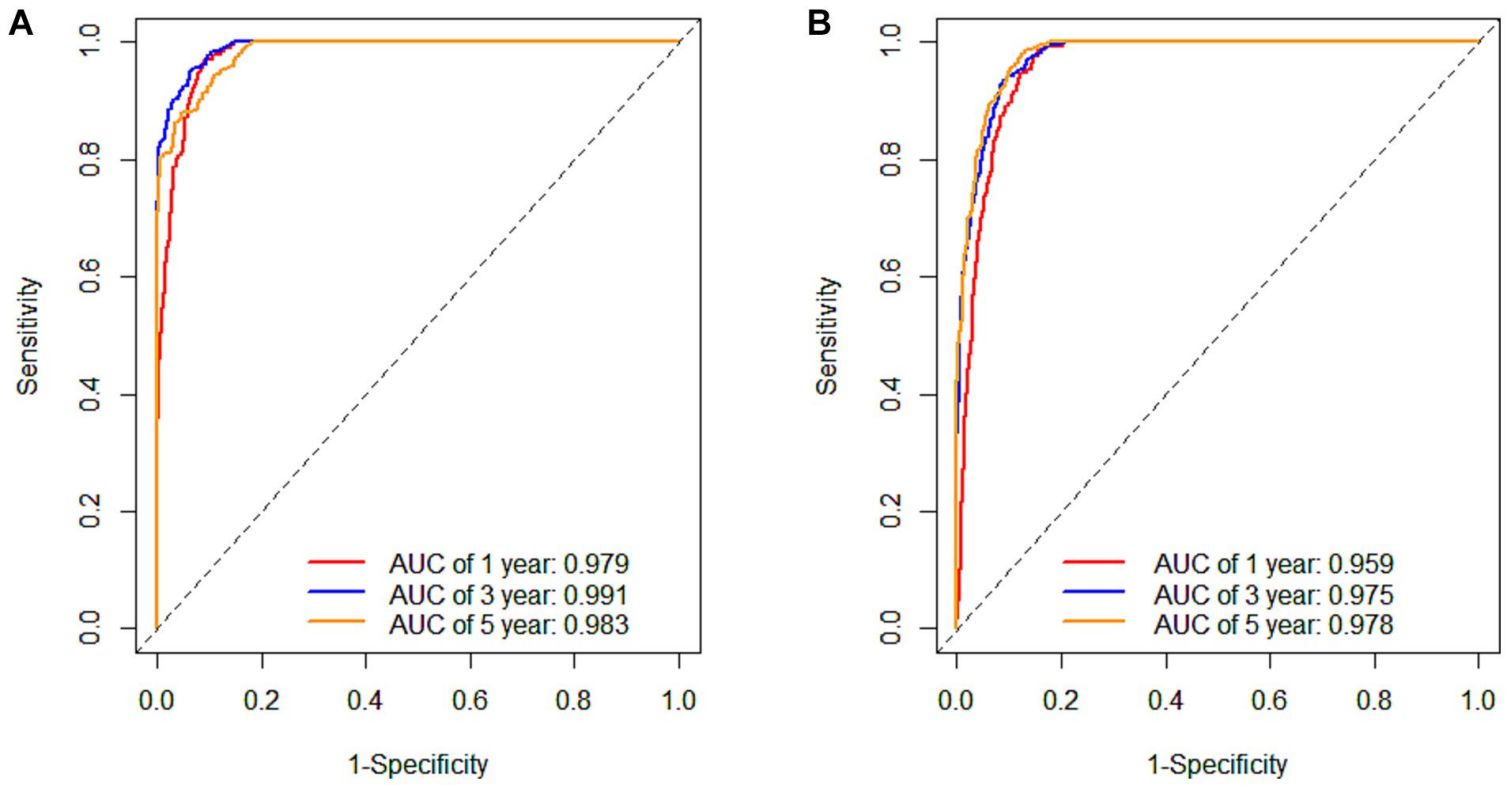
The 1-year, 3-years, and 5-years tdROC curves for internal validation data under the male **(A)** and female **(B)** cohort RSF models.

The tdROC curves for 1 year, 3 years and 5 years were plotted for the male and female cohort models separately (Figure 4). The AUCs of the male cohort model were 0.979, 0.991 and 0.983, indicating the accuracy of the model in predicting the occurrence of MetS at 1 year, 3 years and 5 years were 97.9%, 99.1% and 98.3%, respectively. The AUCs of the female cohort model were 0.959, 0.975 and 0.978, indicating the accuracy of the model in predicting the occurrence of MetS at 1-year, 3-years, and 5-years were 95.9%, 97.5% and 97.8%, respectively. Table 4 shows that the C-indexes calculated by 500 bootstraps of the male and female cohort RSF models is above 0.7, indicating good prediction performance of the model. Overall, the male and female cohort prediction models have demonstrated discrimination ability.

**Table 4:** The C-indexes and prediction error rate calculated by 500 bootstraps of the male and female cohort RSF models.

	C-indexes	Prediction error rate (1-C-indexes)
Male cohort	0.7928	0.2072
Female cohort	0.8528	0.1472

#### 3.4.2 External validation

In this study, the health examination data from Dalian Central Hospital were used to externally verify the constructed model. According to the inclusion and exclusion criteria, a total of 1939 eligible subjects were enrolled into the external validation cohort. There were 324 (16.71%) male with the median baseline age of 33 (30, 31) years, and the median follow-up time of 2.97 (1.05, 5.10) years. In male cohort, 94 subjects occurred MetS, then the incidence rate was 29.01%, and the incidence density was 7.73/100 person-years. There were 1,615 (83.29%) female, with the median age baseline of 28 (32, 33) years, and the median follow-up time of 5.94 (2.92, 8.04) years. In female cohort, 402 subjects occurred MetS, then the incidence rate was 24.89%, and the incidence density was 4.44 per 100 person-years. Details are shown in Supplementary Table S12 and S13.

**Figure 5:**
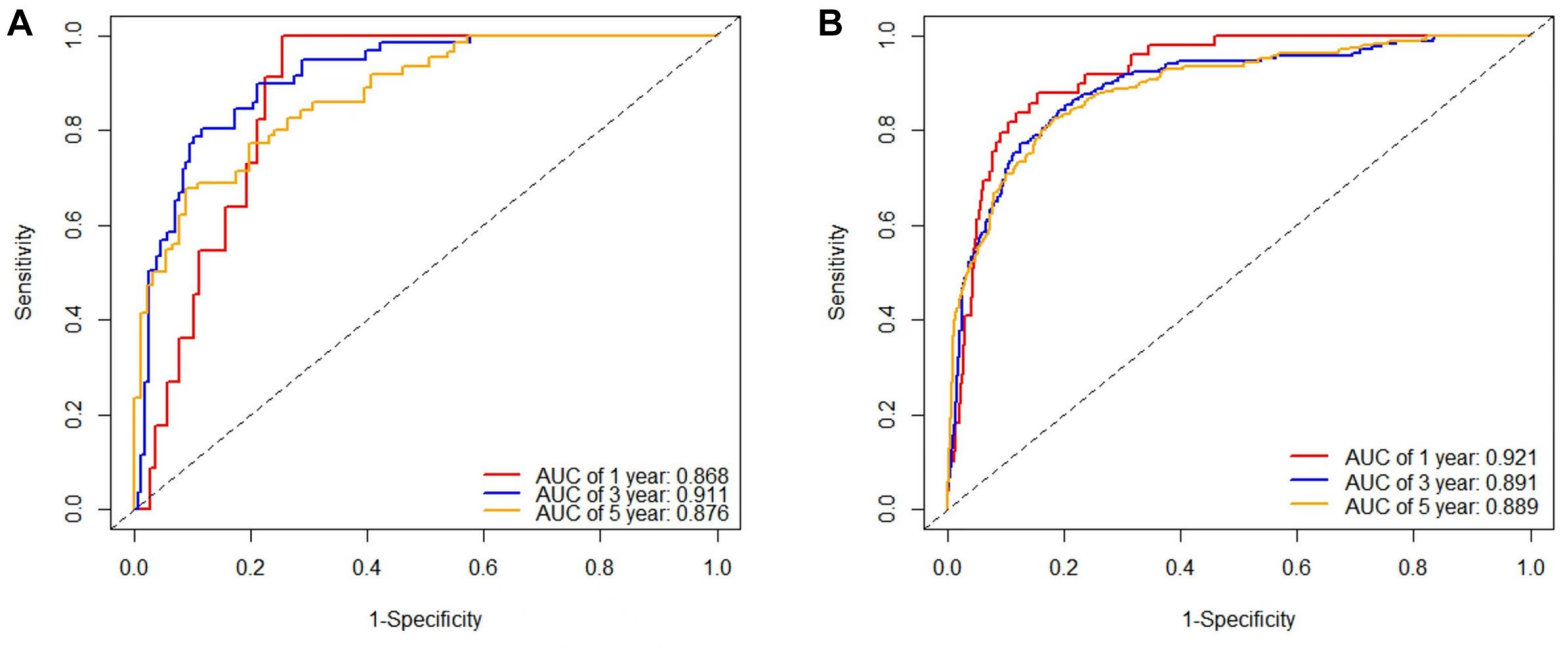
The 1-year, 3-years, and 5-years tdROC curves for external validation data under the male **(A)** and female **(B)** cohort RSF models.

Similarly to the internal validation, tdROC curves were drawn for 1 year, 3 years and 5 years for male and female cohorts separately (Figure 5). The AUCs of the male cohort model were 0.868, 0.911 and 0.876, and the AUCs of the female cohort model were 0.921, 0.891 and 0.889, indicating that the model still had good predictive ability for individuals in the external data.

### 3.5 MetS risk prediction tool

**Figure 6:**
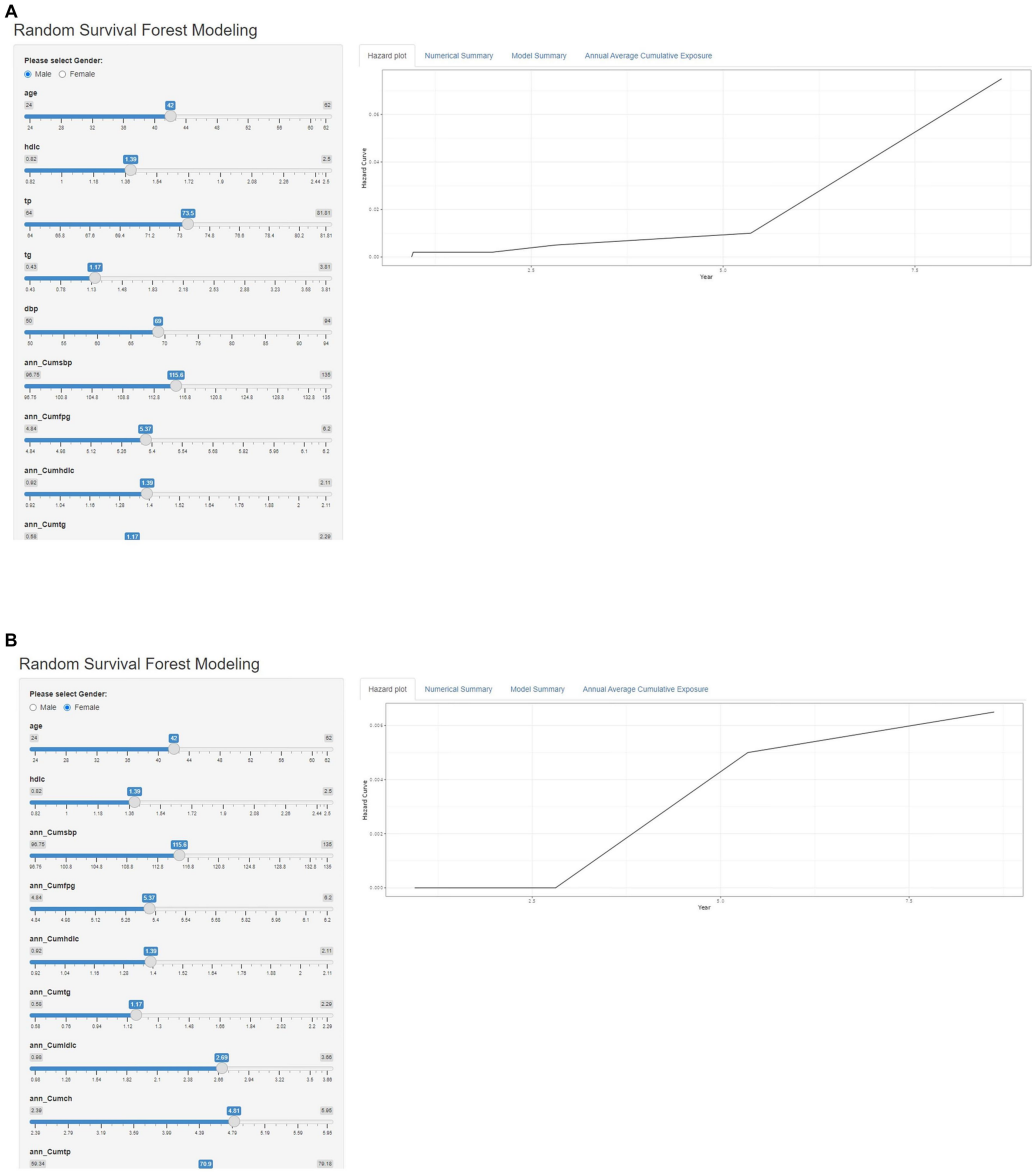
Interface diagram of the MetS risk web prediction calculator for males **(A)** and females **(B)**.

In this study, the constructed risk prediction model was converted into a web-based prediction calculator for MetS risk, and the web-based prediction calculators for MetS risk for male and female can be accessed at https://liuwenxi990903.shinyapps.io/app_04/. The interface of the Web Prediction Calculator is shown in Figure 6. Entering relevant physical examination metrics and reading output numbers and graphs generated by the web server can easily help researchers and clinicians predict and monitor the risk of MetS.

## 4 Discussion

MetS is a systemic disorder that combines a variety of metabolic disorders or diseases (34). With the progress and development of society, the change of residents’ diet structure and living behavior, and the increase of obesity rate, the prevalence of MetS is increasing year by year, and it is showing a younger trend. MetS populations are high-risk groups and subclinical patients with chronic diseases such as cardiovascular disease and diabetes, and if not controlled early, they will cause great harm to human health (28, 32). Therefore, in-depth understanding of the risk factors for the occurrence of MetS, accurate identification of high-risk groups of MetS, formulation of interventions, and early individualized prevention and treatment are essential to prevent the occurrence of cardiovascular and cerebrovascular diseases and type 2 diabetes. It’s very necessary to carry out research on MetS risk prediction models based on domestic populations.

With the rapid development of computer technology, the continuous improvement of hardware performance, informatization is the general direction of development in today’s world. Machine learning as a popular discipline in recent years has played an important role in the field of medicine, the purpose is to use a variety of data to train models, through continuous improvement of algorithms to improve the analysis and prediction ability of models (24). Compared to traditional methods, machine learning is believed to be able to build more efficient and convenient predictive models. In many areas, machine learning has achieved widespread application and has shown great value. Survival analysis, which is closely related to patient prognosis prediction, is a large branch of statistics, of which the Cox proportional hazards regression model is the most commonly used means of survival analysis (30). In the tide of machine learning, some scholars combine machine learning models with survival analysis, and RSF is one of the most representative methods. RSF is a collection of trees and is a nonparametric method constructed by packing a classification tree of right-censored data (26, 35). It is suitable for high-dimensional data where the number of covariates exceeds the number of observations, in addition, it can also work with data consisting of complex and nonlinear relationships between dependent and independent variables.

How to efficiently and accurately screen predictors is the top priority of model construction. In the construction of MetS risk prediction model based on physical examination population in Taiwan by Yang (11) et al. and the study of Hsiao et al. (36), stepwise logistic regression method was used to screen the variables in the model and construct the prediction model. In the MetS risk prediction model based on Shandong multi-center health management population by Sun et al. (32), a multivariate Cox regression method was used to screen variables and construct a prediction model. The above domestic studies have adopted relatively single methods for screening predictors, which has certain limitations. In view of this situation, three methods were used to preliminarily screen the predictors by using the univariate Cox model, the Lasso-Cox model based on the Lasso algorithm and the RSF algorithm.

In this study cohort, the incidence density of the male cohort was 16.71/100 person-years and the female cohort was 8.15/100 person-years, and there were some differences between different sexes. Moreover, in the study indicators comparing the MetS population with the non-MetS population in the male and female cohort, there were slightly different results for the difference in the study indicators. This may be related to the different sample sizes of male and female in the cohort, and the differences between male and female in genetics, endocrine metabolism, intrinsic predisposition, and risk factor exposure. Therefore, this study analyzes male and female separately. Firstly, the three methods of univariate Cox model, Lasso-Cox model based on Lasso algorithm and RSF algorithm were used to initially screen the predictors. As one of the most important analysis methods in survival analysis, the univariate Cox model is widely used in risk factor screening and prediction of clinical follow-up data (37), but it cannot solve the multicollinearity problem, and the screened predictors are not completely reliable. Lasso regression is a statistical inference method for linear regression models of high-dimensional data, which is widely applied to the Cox proportional hazards regression model for survival analysis of high-dimensional data (38). The Lasso-Cox model based on Lasso algorithm can screen out the set of independent variables with strong explanatory power for the dependent variable, while avoiding the multicollinearity problem, which is helpful to improve the accuracy of the risk prediction model (39). However, the limitation is that when the independent variables are highly collinear or highly correlated, Lasso may forcibly delete an independent variable, which will lose the predictive power of the model. The RSF algorithm is a derivative of the random forest algorithm in survival analysis, and its random characteristics process medical data with high-dimensional complex characteristics and are not easy to overfit, and have the advantages of good classification, prediction and complex relationships between analysis variables (33). However, the limitation of the RSF algorithm is that it is poorly visualized and cannot explain the effect of independent variables on the dependent variable (40). In this study, the univariate Cox model, the Lasso-Cox model based on the Lasso algorithm and the RSF algorithm were combined to screen important physical examination indicators, and achieved complementary advantages. The predictors screened by the three methods were summarized, and 19 predictors such as AGE, BMI, SBP, DBP were screened out in the male cohort, and 14 predictors such as AGE, SBP, FPG, HDLC were screened out in the female cohort. In order to ensure the accuracy of the risk prediction model, the Cox stepwise regression method (forward method, backward method, forward backward hybrid method) and optimal subset regression method were used to screen the common predictors further. Finally, the male cohort included a total of 15 predictors in the model: AGE, DBP, TG, HDLC, TP, ann_CumSBP, ann_CumDBP, ann_CumFPG, ann_CumTG, ann_CumHDLC, ann_CumLDLC, ann_CumAST, ann_CumGGT, ann_CumCH, ann_CumRBC; the female cohort included a total of 9 predictors in the model: AGE, HDLC, ann_CumSBP, ann_CumFPG, ann_CumTG, ann_CumHDLC, ann_CumLDLC, ann_CumCH, and ann_CumTP. The most important difference in the predictors screened out by the male and female cohorts was that the male cohort had four more variables: ann_CumAST, ann_CumGGT, ann_CumDBP, and ann_CumRBC than the female cohort. Combined with the comparison of the incidence density of MetS in the male and female cohorts in this study and the results of the current relevant studies (41–43), this difference may be due to the fact that the pathogenesis, risk factors, and distribution of MetS components differ in both male and female populations. In addition, greatly different sample sizes in male and female groups in this study might be another reason. Therefore, we need to consider the above situation when selecting indicators for male and female MetS risk prediction models. In the future, the effectiveness of predictor screening will also need to be validated in larger samples.

At present, a variety of risk factors related to the incidence of MetS have been found in domestic and foreign studies (25, 29, 31, 44, 45), and this study used health management longitudinal physical examination data, conventional biochemical indicators and disease history data to construct MetS risk prediction models for male and female cohorts, respectively. Among them, FPG, TG, HDLC, LDLC, SBP, and DBP, as components and diagnostic-related indicators of MetS, have been confirmed by studies to be influencing factors of MetS (27, 46–49). Most of the predictors included in the model in this study are consistent with this. However, this study differs from previous studies in that we focus not only on the baseline levels of physical examination indicators such as blood lipids, blood glucose, blood pressure, triglycerides, and total cholesterol, but more importantly, on its annual average cumulative exposure. In fact, with the change of people’s living environment and the influence of work, medical treatment, eating habits and other factors, the individual’s physical examination indicators may not always be maintained at the baseline level, but will change over time, so the previous results may lack practicality and are not convincing. Therefore, the results of this study not only enhances the persuasiveness of previous research results, but also prompts us to update our concept and not ignore the dynamic changes of physical examination indicators over time and long-term cumulative exposure when constructing MetS risk prediction models for healthy physical examination populations. Regular physical examination in daily life, more attention to the long-term dynamic changes in these indicators, help identify MetS high-risk groups, provide them with health guidance, guide them to change their diet and living habits in time, actively participate in physical activities, and control the continuous growth of these indicators, which can effectively reduce the risk of MetS or slow down its progression, so as to achieve early prevention of chronic diseases.

In the process of comparing the Cox proportional hazards regression model with the RSF model, it is found that the RSF model has better discrimination and calibration from the perspective of prediction error curve and integrated Brier score. In addition, the Schoenfeld residual method was used to test the PH hypothesis of Cox proportional hazards regression model, and it was found that some independent variables did not meet the PH hypothesis in both male and female cohorts, which further confirmed that the use of the RSF model to construct risk prediction models is more reliable. It can be seen that in the construction of risk prediction model based on large sample longitudinal health examination data, the RSF model has more advantages, which also shows that machine learning has relative advantages over traditional methods, which provides new ideas and directions for the construction of risk prediction models.

The ranking of variable importance given by the constructed RSF model suggests that we should focus on the cumulative exposure of blood lipids, blood glucose, and blood pressure, especially blood glucose. Current studies generally believe that MetS is characterized by a combination of central obesity, diabetes mellitus or impaired glucose tolerance, hypertension, dyslipidemia and hyperuricemia, which is determined by genetic factors and environmental factors (50). Abdominal obesity, chronic subclinical inflammation and the resulting insulin resistance (IR) are the main pathophysiological basis of MetS, which is the central link (51, 52). IR can also lead to vasoconstriction and sodium retention, resulting in increased blood pressure (53), and patients with hypertension and abnormal glucose metabolism often have coexisting abnormal factors of lipid metabolism (52). Therefore, these three indicators are interrelated and cause and effect of each other, which together lead to the occurrence and development of MetS.

In this study, the time-dependent receiver operating characteristic curve (tdROC) was used to evaluate the model, and it was generally believed that the larger the area under the curve (AUC), the better the discrimination of the model. The internal and external validation results of the model show that the predictive performance of the model is good and shows good generalization ability in both male and female cohorts. Internal validation of the RSF model showed that the area under the curve (AUC) of tdROC for 1 year, 3 years and 5 years in the male cohort were 0.979,0.991 and 0.983, the AUC area in the 3 years is higher than in the 5 years, indicating that the male MetS risk prediction model has better discrimination ability than 5 years at the time point of 3 years; the area under the curve (AUC) of tdROC for 1 year, 3 years and 5 years in the female cohort were 0.959, 0.975 and 0.978, there is a gradual increasing trend, indicating that the discrimination ability of the model is also increasing year by year. This may be related to the median follow-up time of 1.39 (0.91, 4.01) years in the male cohort and 3.01 (1.12, 5.03) years in the female cohort. Most of the male’s health check-up population did not reach five years of follow-up, and the female’s health check-up population did reach five years, so this also indirectly affected the differentiation of the model.

In this study, a web prediction calculator for MetS risk was designed by sex based on the RSF model. For clinical application, the predictors included in the model, whether male or female cohorts, are routine indicators of health examination, and the detection cost is low, which is common in clinical practice, and is easy to obtain, which has good clinical application value (12, 54).

## 5 Conclusion

In conclusion, in the construction of MetS risk prediction model based on large sample longitudinal health examination data, the RSF model is better than the Cox proportional hazards regression model. Moreover, the RSF model can concisely and accurately measure the contribution of each predictor to the model, and both males and females should focus on the cumulative exposure of blood lipids, blood glucose and blood pressure, especially the influence of blood glucose on the risk of Mets. The MetS random survival forest risk prediction models for males and females constructed based on multiple screening routine physical examination indicators performed well in internal and external data and had certain clinical application value.

## 6 Strengths and limitations

The advantage of this study is that it makes full use of the abundant health examination information and widely includes physical examination indicators that may be related to the incidence of MetS on the basis of previous studies, considering both their baseline levels and their cumulative exposures. Focusing on long-term dynamic changes, indicators are readily available, and a variety of methods are used to screen the predictors of MetS, which improves the predictive ability of the MetS risk prediction model built on this basis. Compared with the traditional method, the RSF modeling method can automatically evaluate the complex influence and interaction between predictors from an objective perspective under the condition that the PH hypothesis is not met, and rank it based on the importance value output of the model. Finding influential covariates by RSF can also reduce generalization errors, and have better risk prediction than traditional models. In this study, a simple prediction tool, the MetS Risk Prediction Calculator, was established to help clinicians more easily predict and monitor the risk of MetS.

However, there are some limitations in this study. The sample population of this study is only from Dalian City, and the male cohort sample size is small, which makes the extrapolation of the results of the study limited. The physical examination data information is not perfect, and the follow-up time is relatively short, and follow-up research is still needed to further verify the accuracy and effectiveness of the risk assessment model. The predictors included in this study are readily available, but relatively traditional, and it is necessary to include more new and sensitive predictors in future studies. The lifestyle factors such as eating habits and physical activity, lack in the physical examination data, should also be considered in future studies. Finally, in this study, the first web prediction calculator specially designed for MetS was established, but the design of the web prediction calculator is more flexible and ever-changing, and new design schemes are constantly proposed, which need further optimization in practical applications.

## Data availability statement

The raw data supporting the conclusions of this article will be made available by the authors, without undue reservation.

## Ethics statement

The studies involving human participants were reviewed and approved by Ethics Committee of Dalian Medical University (Ethics approval no. 2020 006). Written informed consent for participation was not required for this study in accordance with the national legislation and the institutional requirements.

## Author contributions

WL: Conceptualization, Formal analysis, Investigation, Methodology, Resources, Software, Validation, Visualization, Writing – original draft, Writing – review & editing. XT: Data curation, Writing – review & editing. TC: Data curation, Writing – review & editing. HZ: Data curation, Writing – review & editing. GS: Conceptualization, Funding acquisition, Methodology, Project administration, Supervision, Writing – review & editing.
