# Supplementary material for: Development and visualization of a risk prediction model for metabolic syndrome: a longitudinal cohort study based on health check-up data in China
Source: Front Nutr. 2023 Nov 21;10:1286654. doi: 10.3389/fnut.2023.1286654 (PMC10702500; doi:10.3389/fnut.2023.1286654)
Supplement: Supplementary file 1 [file Data_Sheet_1.docx]

Supplementary Material

**Development and visualization of a** **risk prediction model for metabolic syndrome: A longitudinal cohort study based on health check-up data in China**

Wenxi Liu^1^, Xiao Tang^1^, Tongcheng Cui^1^, Hui Zhao ^2^ and Guirong Song^1*^

*** Correspondence:** Guirong Song: [songsara2016@dmu.edu.cn](mailto:songsara2016@dmu.edu.cn)

# Supplementary Figures and Tables

## Supplementary Figures

Data on health check-ups from January 2011 to December 2021 at the health check-ups centre of the Second Hospital Affiiated with Dalian Medical University in Dalian were obtained.

the baseline age≤60 years old.

no a diagnosis of MetS at baseline.

no history of cardiovascular and cerebrovascular diseases, diabetes, liver disease or renal disease at baseline.

no absence of baseline MetS diagnostic information.

the baseline cohort (n=5691).

missing data related to MetS components, or were diagnosed with cardiovascular and cerebrovascular diseases, diabetes and other diseases during follow-up (n=162).

not undergo follow-up (n=74).

the female valid analysis cohort (n=3928).

the male valid analysis cohort (n=1527).

the valid analysis cohort (n=5455).

**Figure 1.** The process of the valid cohort establishment.

**Measurements of indicator**

**a2**

**a1**

**Duration of follow-up**

**t2**

**t1**

**Figure 2.** Schematic diagram of the area under the curve of two adjacent physical examinations for an indicator.


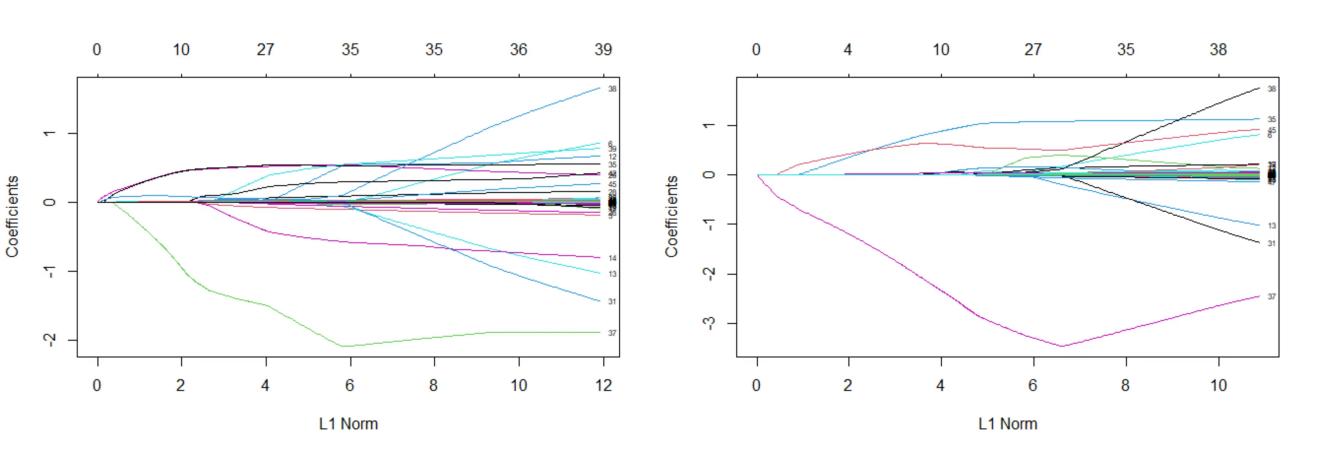


B

A


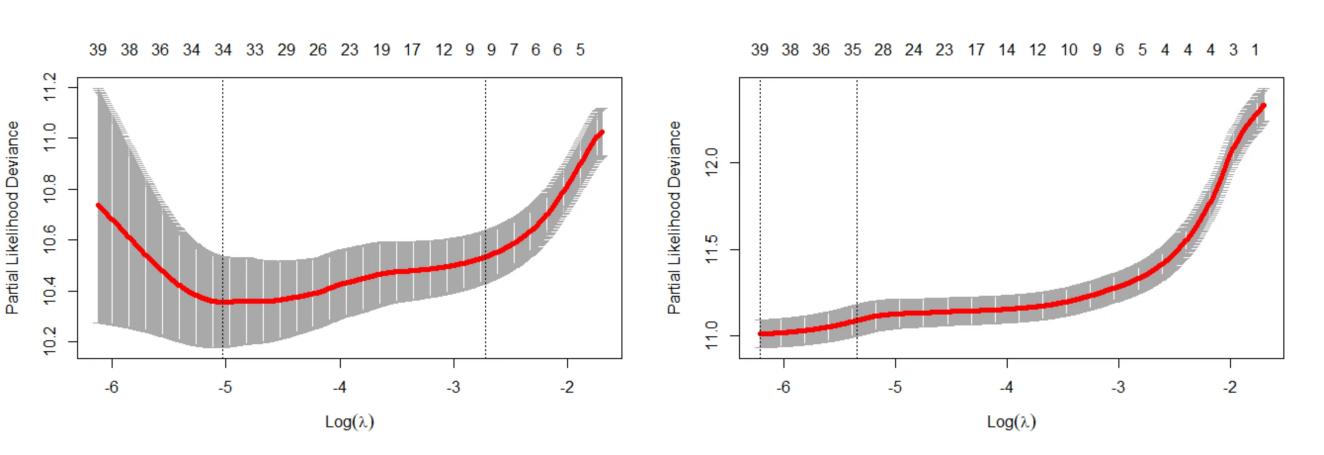


D

C

**Figure 3.** LASSO-Cox regression plot. **(A)** Plot of partial likelihood deviance of the male cohort. **(B)** Plot of partial likelihood deviance of the female cohort. **(C)** Plot of LASSO coefficient profiles of the male cohort. **(D)** Plot of LASSO coefficient profiles of the female cohort.


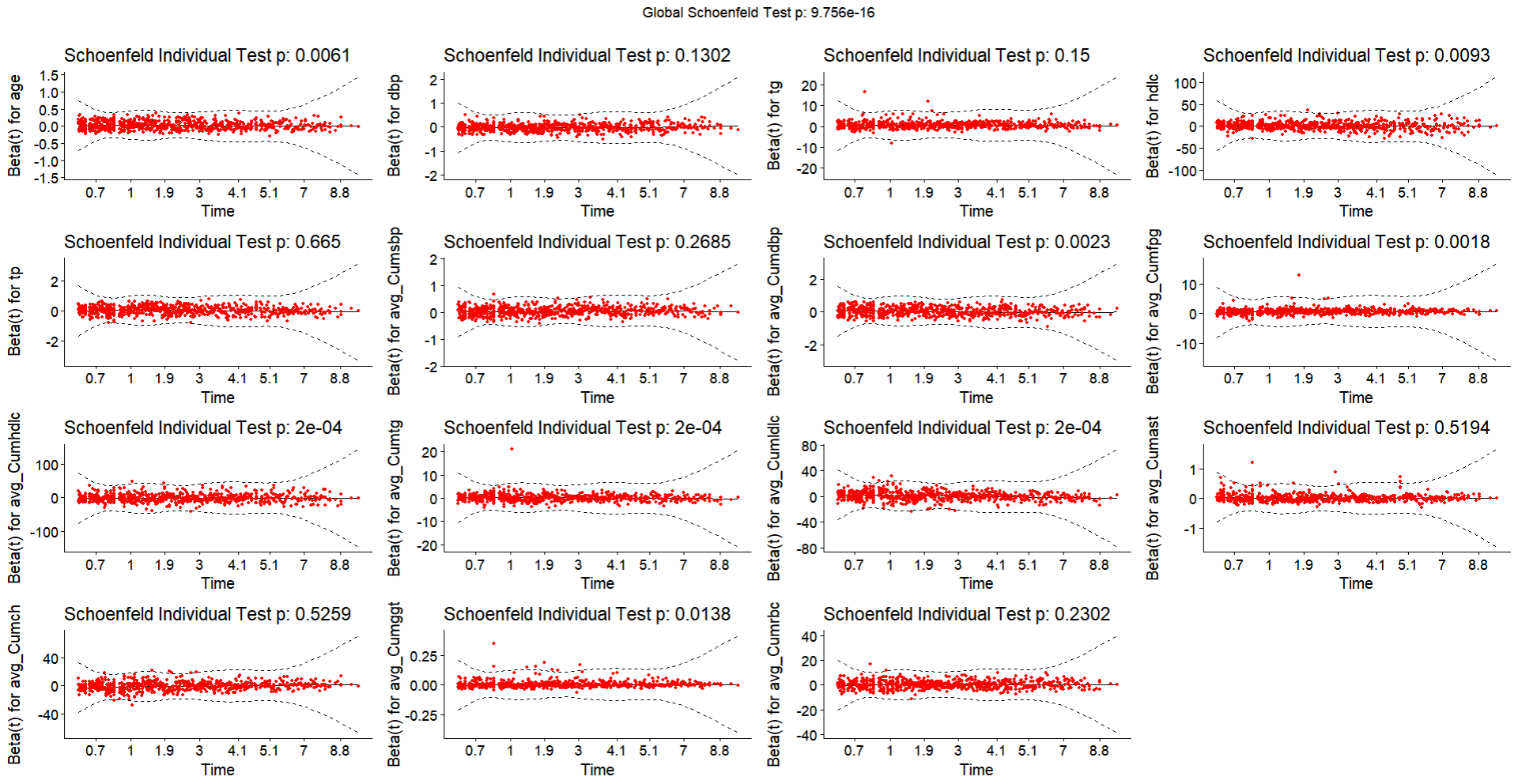


A


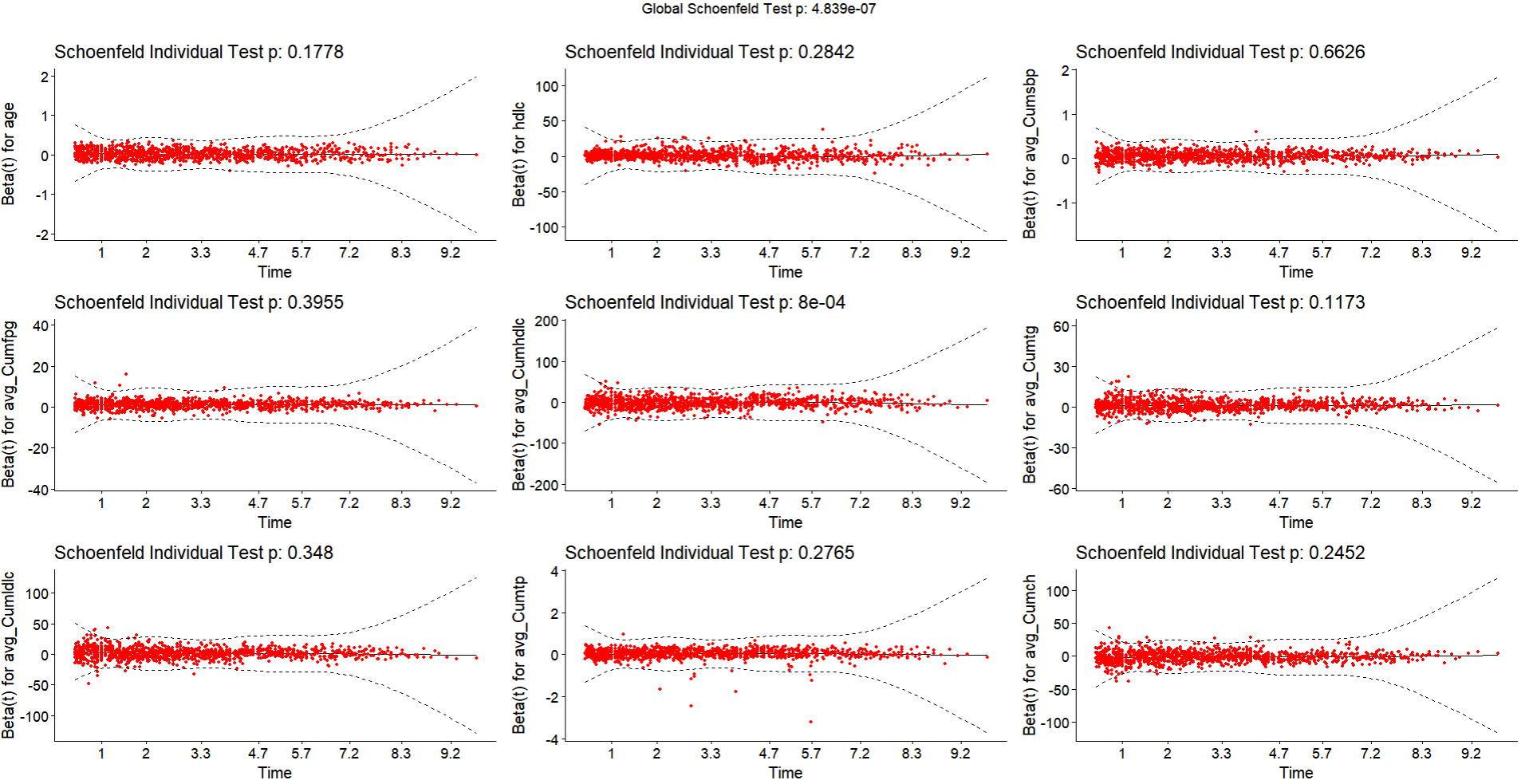


B

**Figure 4.** Schoenfeld residual plot of the Cox proportional hazards regression model. **(A)** Schoenfeld residual plot of the male cohort. **(B)** Schoenfeld residual plot of the female cohort.

## Supplementary Tables

**Table 1.** A basic description of the variables in this study.

| **Variables** | | **Variable Description** |
| --- | --- | --- |
| **Outcome Events** | MetS | Whether MetS occurs, MetS is "0" if it does not occur, and "1" if it occurs |
| **Times** | year | Years of follow-up |
| **Predictors** | Age (years) | Baseline Age |
|  | BMI (kg/m^2^) | Body Mass Index |
|  | SBP (mmHg) | Systolic Blood Pressure |
|  | DBP (mmHg) | Diastolic Blood Pressure |
|  | FPG (mmol/L) | Fasting Plasma Glucose |
|  | TG (mmol/L) | Triglycerides |
|  | HDL-C (mmol/L) | High Density Lipoprotein Cholesterol |
|  | LDL-C (mmol/L) | Low Density Lipoprotein Cholesterol |
|  | DBIL (μmol/L) | Direct Bilirubin |
|  | TBIL (μmol/L) | Total Bilirubin |
|  | ALB (g/L) | Albumin |
|  | AST (U/L) | Aspartate Aminotransferase |
|  | ALT (U/L) | Alanine Aminotransferase |
|  | GGT (U/L) | Glutamyltransferase |
|  | CH (mmol/L) | Total Cholesterol |
|  | CR (μmol/L) | Creatinine |
|  | HB (g/L) | Hemoglobin |
|  | LYM (g/L) | Lymphocyte |
|  | NEU (g/L) | Neutrophils |
|  | RBC (g/L) | Red Blood Cell |
|  | WBC (g/L) | White Blood Cell |
|  | PLT (/L) | Blood Platelet |
|  | TP (g/L) | Total Protein |
|  | UA (μmol/L) | Uric Acid |
|  | UREA (mmol/L) | Urea |
|  | ann_CumBMI (kgyear/m^2^) | Annual mean cumulative exposure of body mass index |
|  | ann_CumSBP (mmHgyear) | Annual mean cumulative exposure of systolic blood pressure |
|  | ann_CumDBP (mmHgyear) | Annual mean cumulative exposure of diastolic blood pressure |
|  | ann_CumFPG (mmolyear/L) | Annual mean cumulative exposure of fasting plasma glucose |
|  | ann_CumTG (mmolyear/L) | Annual mean cumulative exposure of triglycerides |
|  | ann_CumHDL-C (mmolyear/L) | Annual mean cumulative exposure of high density lipoprotein cholesterol |
|  | ann_CumLDL-C (mmolyear/L) | Annual mean cumulative exposure of low density lipoprotein cholesterol |
|  | ann_CumDBIL (μmolyear/L) | Annual mean cumulative exposure of direct bilirubin |
|  | ann_CumTBIL (μmolyear/L) | Annual mean cumulative exposure of total bilirubin |
|  | ann_CumALB (gyear/L) | Annual mean cumulative exposure of albumin |
|  | ann_CumAST (Uyear/L) | Annual mean cumulative exposure of aspartate aminotransferase |
|  | ann_CumALT (Uyear/L) | Annual mean cumulative exposure of alanine aminotransferase |
|  | ann_CumGGT (Uyear/L) | Annual mean cumulative exposure of glutamyltransferase |
|  | ann_CumCH (mmolyear/L) | Annual mean cumulative exposure of total cholesterol |
|  | ann_CumCR (μmolyear/L) | Annual mean cumulative exposure of creatinine |
|  | ann_CumHB (gyear/L) | Annual mean cumulative exposure of hemoglobin |
|  | ann_CumLYM (gyear/L) | Annual mean cumulative exposure of lymphocytes |
|  | ann_CumNEU (gyear/L) | Annual mean cumulative exposure of neutrophils |
|  | ann_CumRBC (gyear/L) | Annual mean cumulative exposure of red blood cell |
|  | ann_CumWBC (gyear/L) | Annual mean cumulative exposure of white blood cell |
|  | ann_CumPLT (year/L) | Annual mean cumulative exposure of blood platelet |
|  | ann_CumTP (gyear/L) | Annual mean cumulative exposure of total protein |
|  | ann_CumUA (μmolyear/L) | Annual mean cumulative exposure of uric acid |
|  | ann_CumUREA (mmolyear/L) | Annual mean cumulative exposure of urea |

**Table 2.** The comparison of potential predictor variables between MetS group and non-MetS group in male cohort.

| **Variables** | **MetS（n=671）** | | **Non-MetS（n=856）** | | ***Z*  *P*** | |
| --- | --- | --- | --- | --- | --- | --- |
|  | **M P_25_，P_75_** | | **M P_25_，P_75_** | |  |  |
| AGE | 40.00 | 30.50,47.00 | 36.00 | 29.00,47.00 | 18.84 | 0.001 |
| BMI | 24.49 | 22.89,26.33 | 23.67 | 21.67,25.62 | 20.46 | ＜0.001 |
| SBP | 123.00 | 116.00,129.00 | 120.00 | 112.00,128.00 | 19.44 | ＜0.001 |
| DBP | 76.00 | 70.00,81.00 | 73.00 | 67.00,78.00 | 20.50 | ＜0.001 |
| FPG | 5.42 | 5.21,5.59 | 5.30 | 5.07,5.51 | 20.56 | ＜0.001 |
| TG | 1.21 | 0.93,1.49 | 1.00 | 0.77,1.28 | 21.44 | ＜0.001 |
| HDLC | 1.18 | 1.05,1.36 | 1.26 | 1.13,1.43 | 13.57 | ＜0.001 |
| LDLC | 2.87 | 2.45,3.40 | 2.63 | 2.24,3.07 | 20.48 | ＜0.001 |
| DBIL | 4.60 | 3.70,6.09 | 4.92 | 3.89,6.32 | 15.90 | 0.010 |
| TBIL | 14.30 | 11.10,18.16 | 14.60 | 11.50,18.82 | 16.49 | 0.161 |
| ALB | 47.20 | 45.72,49.00 | 47.32 | 45.79,49.15 | 16.82 | 0.464 |
| AST | 22.00 | 19.00,26.99 | 21.00 | 18.00,25.00 | 18.82 | 0.001 |
| ALT | 24.04 | 18.00,34.38 | 21.00 | 16.00,29.45 | 20.03 | ＜0.001 |
| GGT | 24.00 | 17.00,34.85 | 19.19 | 14.52,27.25 | 20.75 | ＜0.001 |
| CH | 4.77 | 4.24,5.28 | 4.62 | 4.13,5.12 | 18.93 | 0.001 |
| CR | 78.00 | 71.73,84.93 | 78.84 | 72.00,86.00 | 16.61 | 0.247 |
| HB | 155.00 | 148.00,161.00 | 154.70 | 148.00,160.00 | 17.84 | 0.188 |
| LYM | 2.17 | 1.82,2.57 | 2.02 | 1.69,2.43 | 19.55 | ＜0.001 |
| NEU | 3.35 | 2.81,4.21 | 3.27 | 2.66,3.93 | 18.67 | 0.003 |
| RBC | 5.11 | 4.85,5.33 | 5.08 | 4.85,5.28 | 17.88 | 0.163 |
| WBC | 6.20 | 5.29,7.15 | 5.86 | 5.03,6.83 | 19.44 | ＜0.001 |
| PLT | 216.00 | 190.00,247.00 | 216.50 | 190.00,246.00 | 17.21 | 0.965 |
| TP | 72.40 | 69.69,75.40 | 72.93 | 70.37,75.69 | 16.01 | 0.018 |
| UA | 382.00 | 330.50,428.01 | 372.07 | 327.98,417.96 | 18.22 | 0.036 |
| UREA | 4.99 | 4.33,5.80 | 5.06 | 4.35,5.83 | 16.77 | 0.399 |
| ann_CumBMI | 24.86 | 23.31,26.55 | 23.85 | 21.91,25.61 | 21.04 | ＜0.001 |
| ann_CumSBP | 126.00 | 119.85,132.05 | 120.50 | 114.61,126.77 | 22.60 | ＜0.001 |
| ann_CumDBP | 77.50 | 72.75,82.50 | 72.50 | 68.00,7.18 | 23.30 | ＜0.001 |
| ann_CumFPG | 5.49 | 5.26,5.69 | 5.29 | 5.11,5.46 | 23.09 | ＜0.001 |
| ann_CumTG | 1.38 | 1.12,1.70 | 1.10 | 0.88,1.30 | 24.12 | ＜0.001 |
| ann_CumHDLC | 1.18 | 1.05,1.34 | 1.28 | 1.15,1.45 | 12.51 | ＜0.001 |
| ann_CumLDLC | 2.80 | 2.39,3.28 | 2.66 | 2.26,3.08 | 19.80 | ＜0.001 |
| ann_CumDBIL | 4.69 | 3.79,5.96 | 5.05 | 4.04,6.26 | 15.20 | ＜0.001 |
| ann_CumTBIL | 14.50 | 11.77,17.89 | 15.23 | 12.39,18.68 | 15.65 | 0.002 |
| ann_CumALB | 47.28 | 46.03,48.68 | 47.37 | 45.94,48.94 | 16.94 | 0.625 |
| ann_CumAST | 22.15 | 19.54,25.98 | 20.60 | 18.00,23.54 | 20.57 | ＜0.001 |
| ann_CumALT | 25.61 | 19.96,35.32 | 21.58 | 16.53,28.90 | 21.40 | ＜0.001 |
| ann_CumGGT | 24.73 | 18.00,35.50 | 19.47 | 14.85,27.40 | 21.61 | ＜0.001 |
| ann_CumCH | 4.86 | 4.36,5.36 | 4.67 | 4.27,5.18 | 19.25 | ＜0.001 |
| ann_CumCR | 77.00 | 71.32,84.00 | 77.98 | 71.33,84.10 | 16.48 | 0.156 |
| ann_CumHB | 155.43 | 149.90,161.40 | 154.50 | 149.00,159.50 | 18.52 | 0.007 |
| ann_CumLYM | 2.16 | 1.87,2.61 | 2.01 | 1.70,2.39 | 20.31 | ＜0.001 |
| ann_CumNEU | 3.45 | 2.92,4.13 | 3.27 | 2.75,3.95 | 18.97 | ＜0.001 |
| ann_CumRBC | 5.12 | 4.88,5.34 | 5.08 | 4.86,5.28 | 18.64 | 0.003 |
| ann_CumWBC | 6.21 | 5.43,7.08 | 5.85 | 5.09,6.80 | 19.85 | ＜0.001 |
| ann_CumPLT | 220.07 | 193.50,250.25 | 220.61 | 192.18,250.50 | 17.22 | 0.941 |
| ann_CumTP | 73.30 | 70.93,75.51 | 73.08 | 70.61,75.70 | 17.64 | 0.354 |
| ann_CumUA | 382.54 | 338.95,425.92 | 374.42 | 332.82,415.18 | 18.50 | 0.008 |
| ann_CumUREA | 5.12 | 4.53,5.76 | 5.18 | 4.55,5.81 | 16.74 | 0.375 |

**Table 3.** The comparison of potential predictor variables between MetS group and non-MetS group in female cohort.

| **Variables** | **MetS（n=1115）** | | **Non-MetS（n=2813）** | | ***Z*  *P*** | |
| --- | --- | --- | --- | --- | --- | --- |
|  | **M P_25_，P_75_** | | **M P_25_，P_75_** | |  |  |
| AGE | 35.00 | 28.00,43.00 | 31.00 | 26.00,39.00 | 36.51 | ＜0.001 |
| BMI | 22.31 | 20.57,24.14 | 21.09 | 19.63,22.86 | 37.54 | ＜0.001 |
| SBP | 118.00 | 110.00,125.00 | 113.00 | 106.00,121.00 | 36.72 | ＜0.001 |
| DBP | 72.00 | 66.00,78.00 | 69.00 | 63.00,75.00 | 36.40 | ＜0.001 |
| FPG | 5.25 | 5.00,5.46 | 5.10 | 4.87,5.33 | 36.77 | ＜0.001 |
| TG | 0.93 | 0.70,1.24 | 0.82 | 0.62,1.14 | 35.38 | ＜0.001 |
| HDLC | 1.38 | 1.24,1.56 | 1.55 | 1.38,1.74 | 24.86 | ＜0.001 |
| LDLC | 2.60 | 2.15,3.07 | 2.31 | 1.94,2.75 | 37.30 | ＜0.001 |
| DBIL | 3.70 | 2.85,4.70 | 3.90 | 3.08,5.09 | 29.88 | ＜0.001 |
| TBIL | 11.37 | 9.10,13.93 | 11.97 | 9.72,15.01 | 30.24 | ＜0.001 |
| ALB | 45.90 | 44.20,47.58 | 46.00 | 44.36,47.80 | 31.43 | 0.082 |
| AST | 19.00 | 16.00,22.00 | 18.00 | 16.00,21.00 | 33.42 | 0.008 |
| ALT | 15.00 | 12.00,20.00 | 14.00 | 11.00,18.00 | 35.31 | ＜0.001 |
| GGT | 13.00 | 10.00,17.00 | 11.50 | 9.14,14.00 | 36.33 | ＜0.001 |
| CH | 4.56 | 4.06,5.17 | 4.46 | 4.03,4.97 | 33.87 | ＜0.001 |
| CR | 55.00 | 50.00,61.00 | 55.00 | 50.07,60.92 | 32.22 | 0.992 |
| HB | 133.00 | 127.00,139.00 | 132.00 | 126.00,138.00 | 33.26 | 0.020 |
| LYM | 1.99 | 1.70,2.34 | 1.93 | 1.62,2.29 | 33.90 | ＜0.001 |
| NEU | 3.31 | 2.73,4.13 | 3.19 | 2.57,3.96 | 33.92 | ＜0.001 |
| RBC | 4.48 | 4.30,4.69 | 4.45 | 4.26,4.66 | 33.75 | 0.001 |
| WBC | 5.84 | 5.00,6.80 | 5.58 | 4.81,6.57 | 34.29 | ＜0.001 |
| PLT | 242.00 | 209.00,278.00 | 236.00 | 204.00,272.00 | 33.56 | 0.003 |
| TP | 72.30 | 69.70,75.60 | 73.00 | 70.01,76.00 | 31.08 | 0.012 |
| UA | 259.00 | 224.80,298.43 | 252.51 | 221.35,289.80 | 33.74 | 0.001 |
| UREA | 4.15 | 3.57,4.85 | 4.17 | 3.53,4.89 | 32.14 | 0.857 |
| ann_CumBMI | 22.84 | 21.10,24.62 | 21.45 | 19.93,23.13 | 38.74 | ＜0.001 |
| ann_CumSBP | 121.00 | 114.47,126.50 | 114.62 | 109.00,120.50 | 40.13 | ＜0.001 |
| ann_CumDBP | 73.08 | 68.50,78.02 | 69.21 | 65.00,73.92 | 38.90 | ＜0.001 |
| ann_CumFPG | 5.30 | 5.08,5.52 | 5.10 | 4.91,5.28 | 40.11 | ＜0.001 |
| ann_CumTG | 1.17 | 0.89,1.46 | 0.95 | 0.77,1.16 | 39.58 | ＜0.001 |
| ann_CumHDLC | 1.35 | 1.23,1.53 | 1.57 | 1.42,1.75 | 22.10 | ＜0.001 |
| ann_CumLDLC | 2.57 | 2.17,3.02 | 2.29 | 1.96,2.68 | 37.71 | ＜0.001 |
| ann_CumDBIL | 3.71 | 3.05,4.64 | 4.00 | 3.25,4.94 | 29.72 | ＜0.001 |
| ann_CumTBIL | 11.80 | 9.88,14.27 | 12.53 | 10.40,15.09 | 29.66 | ＜0.001 |
| ann_CumALB | 45.91 | 44.52,47.27 | 45.96 | 44.58,47.37 | 31.67 | 0.227 |
| ann_CumAST | 18.48 | 16.39,21.60 | 18.00 | 15.89,20.48 | 34.34 | ＜0.001 |
| ann_CumALT | 16.00 | 12.80,21.00 | 14.35 | 11.74,18.02 | 36.50 | ＜0.001 |
| ann_CumGGT | 13.50 | 10.65,17.62 | 11.26 | 9.50,14.38 | 37.92 | ＜0.001 |
| ann_CumCH | 4.72 | 4.21,5.28 | 4.58 | 4.15,5.09 | 34.17 | ＜0.001 |
| ann_CumCR | 54.50 | 50.25,59.25 | 54.48 | 50.37,59.47 | 32.09 | 0.779 |
| ann_CumHB | 133.50 | 127.50,138.50 | 132.50 | 126.71,137.50 | 33.76 | 0.001 |
| ann_CumLYM | 2.01 | 1.72,2.33 | 1.92 | 1.64,2.23 | 34.55 | ＜0.001 |
| ann_CumNEU | 3.46 | 2.90,4.07 | 3.24 | 2.71,3.91 | 34.95 | ＜0.001 |
| ann_CumRBC | 4.52 | 4.34,4.69 | 4.46 | 4.28,4.64 | 34.93 | ＜0.001 |
| ann_CumWBC | 5.95 | 5.20,6.78 | 5.64 | 4.89,6.46 | 35.31 | ＜0.001 |
| ann_CumPLT | 250.06 | 217.50,284.23 | 241.72 | 211.99,274.93 | 34.18 | ＜0.001 |
| ann_CumTP | 73.70 | 71.35,76.32 | 73.36 | 70.95,75.80 | 33.73 | 0.001 |
| ann_CumUA | 269.00 | 241.45,304.64 | 259.95 | 230.17,289.34 | 35.10 | ＜0.001 |
| ann_CumUREA | 4.26 | 3.71,4.83 | 4.27 | 3.73,4.85 | 31.96 | 0.568 |

**Table 4.** The univariate Cox regression analysis for MetS potential predictor variables in the male cohort.

| **Variables** | **The Univariate Cox Regression Analysis** | |
| --- | --- | --- |
|  | **HR ( 95% CI)** | ***P*** |
| AGE | 1.02(1.02-1.03) | ＜0.001 |
| BMI | 1.12(1.09-1.14) | ＜0.001 |
| SBP | 1.02(1.02-1.03) | ＜0.001 |
| DBP | 1.03(1.02-1.04) | ＜0.001 |
| FPG | 1.43(1.31-1.56) | ＜0.001 |
| TG | 2.08(1.86-2.33) | ＜0.001 |
| HDLC | 0.33(0.23-0.47) | ＜0.001 |
| LDLC | 1.29(1.16-1.43) | ＜0.001 |
| DBIL | 0.98(0.94-1.02) | 0.253 |
| TBIL | 1.01(0.99-1.02) | 0.372 |
| ALB | 0.96(0.93-0.99) | 0.016 |
| AST | 1.02(1.01-1.03) | ＜0.001 |
| ALT | 1.01(1.00-1.01) | ＜0.001 |
| GGT | 1.01(1.00-1.01) | ＜0.001 |
| CH | 1.30(1.18-1.43) | ＜0.001 |
| CR | 1.00(0.99-1.01) | 0.880 |
| HB | 1.01(1.00-1.02) | 0.006 |
| LYM | 1.14(1.00-1.30) | 0.054 |
| NEU | 1.09(1.02-1.15) | 0.010 |
| RBC | 1.45(1.15-1.82) | 0.002 |
| WBC | 1.08(1.03-1.14) | 0.002 |
| PLT | 1.00(1.00-1.01) | 0.020 |
| TP | 1.02(1.00-1.04) | 0.030 |
| UA | 1.00(1.00-1.01) | ＜0.001 |
| UREA | 1.05(0.98-1.12) | 0.210 |
| ann_CumBMI | 1.12(1.10-1.15) | ＜0.001 |
| ann_CumSBP | 1.04(1.03-1.05) | ＜0.001 |
| ann_CumDBP | 1.06(1.05-1.07) | ＜0.001 |
| ann_CumFPG | 1.58(1.46-1.71) | ＜0.001 |
| ann_CumTG | 1.31(1.25-1.37) | ＜0.001 |
| ann_CumHDLC | 0.15(0.10-0.22) | ＜0.001 |
| ann_CumLDLC | 1.50(1.33-1.69) | ＜0.001 |
| ann_CumDBIL | 0.94(0.90-0.98) | 0.005 |
| ann_CumTBIL | 0.99(0.98-1.01) | 0.225 |
| ann_CumALB | 1.01(0.97-1.04) | 0.671 |
| ann_CumAST | 1.04(1.03-1.04) | ＜0.001 |
| ann_CumALT | 1.02(1.01-1.02) | ＜0.001 |
| ann_CumGGT | 1.01(1.00-1.01) | ＜0.001 |
| ann_CumCH | 1.31(1.18-1.45) | ＜0.001 |
| ann_CumCR | 1.00(0.99-1.01) | 0.970 |
| ann_CumHB | 1.02(1.01-1.03) | ＜0.001 |
| ann_CumLYM | 1.40(1.22-1.60) | ＜0.001 |
| ann_CumNEU | 1.13(1.05-1.22) | 0.001 |
| ann_CumRBC | 1.80(1.42-2.31) | ＜0.001 |
| ann_CumWBC | 1.14(1.08-1.21) | ＜0.001 |
| ann_CumPLT | 1.00(0.99-1.00) | 0.785 |
| ann_CumTP | 1.03(1.01-1.04) | 0.001 |
| ann_CumUA | 1.00(1.00-1.01) | ＜0.001 |
| ann_CumUREA | 1.03(0.95-1.12) | 0.476 |

**Table 5.** The univariate Cox regression analysis for MetS potential predictor variables in the female cohort.

| **Variables** | **The Univariate Cox Regression Analysis** | |
| --- | --- | --- |
|  | **HR ( 95% CI)** | ***P*** |
| AGE | 1.03(1.02-1.04) | ＜0.001 |
| BMI | 1.15(1.13-1.17) | ＜0.001 |
| SBP | 1.03(1.02-1.03) | ＜0.001 |
| DBP | 1.04(1.03-1.04) | ＜0.001 |
| FPG | 2.23(1.92-2.58) | ＜0.001 |
| TG | 1.95(1.78-2.14) | ＜0.001 |
| HDLC | 0.18(0.14-0.23) | ＜0.001 |
| LDLC | 1.41(1.31-1.52) | ＜0.001 |
| DBIL | 0.93(0.89-0.96) | ＜0.001 |
| TBIL | 0.98(0.97-1.00) | 0.007 |
| ALB | 0.98(0.96-1.00) | 0.060 |
| AST | 1.01(1.00-1.02) | 0.089 |
| ALT | 1.01(1.00-1.01) | 0.001 |
| GGT | 1.01(1.01-1.02) | ＜0.001 |
| CH | 1.19(1.11-1.27) | ＜0.001 |
| CR | 1.00(0.99-1.01) | 0.768 |
| HB | 1.01(1.00-1.01) | 0.066 |
| LYM | 1.14(1.02-1.27) | 0.026 |
| NEU | 1.09(1.04-1.14) | ＜0.001 |
| RBC | 1.82(1.49-2.21) | ＜0.001 |
| WBC | 1.08(1.04-1.13) | ＜0.001 |
| PLT | 1.01(1.00-1.01) | ＜0.001 |
| TP | 1.03(1.02-1.05) | ＜0.001 |
| UA | 1.01(1.00-1.01) | ＜0.001 |
| UREA | 1.00(0.95-1.06) | 0.986 |
| ann_CumBMI | 1.17(1.14-1.19) | ＜0.001 |
| ann_CumSBP | 1.06(1.05-1.06) | ＜0.001 |
| ann_CumDBP | 1.07(1.06-1.08) | ＜0.001 |
| ann_CumFPG | 2.43(2.25-2.62) | ＜0.001 |
| ann_CumTG | 3.59(3.24-3.98) | ＜0.001 |
| ann_CumHDLC | 0.05(0.04-0.06) | ＜0.001 |
| ann_CumLDLC | 1.63(1.51-1.77) | ＜0.001 |
| ann_CumDBIL | 0.88(0.85-0.93) | ＜0.001 |
| ann_CumTBIL | 0.96(0.94-0.97) | ＜0.001 |
| ann_CumALB | 1.02(1.00-1.05) | 0.110 |
| ann_CumAST | 1.04(1.02-1.05) | ＜0.001 |
| ann_CumALT | 1.03(1.02-1.03) | ＜0.001 |
| ann_CumGGT | 1.01(1.00-1.01) | ＜0.001 |
| ann_CumCH | 1.14(1.06-1.23) | 0.001 |
| ann_CumCR | 1.01(1.00-1.01) | 0.275 |
| ann_CumHB | 1.01(1.00-1.02) | 0.001 |
| ann_CumLYM | 1.42(1.26-1.61) | ＜0.001 |
| ann_CumNEU | 1.21(1.14-1.28) | ＜0.001 |
| ann_CumRBC | 2.12(1.71-2.64) | ＜0.001 |
| ann_CumWBC | 1.94(1.13-1.24) | ＜0.001 |
| ann_CumPLT | 1.00(1.00-1.01) | ＜0.001 |
| ann_CumTP | 1.05(1.03-1.06) | ＜0.001 |
| ann_CumUA | 1.01(1.00-1.01) | ＜0.001 |
| ann_CumUREA | 0.98(0.91-1.05) | 0.518 |

**Table 6.** The error rates corresponding to different numbers of survival trees in the male cohort and female cohort based on the random survival forest algorithm.

| **n_tree_** | **Error Rates(%)** | |
| --- | --- | --- |
|  | **Male Cohort** | **Female Cohort** |
| 100 | 21.74 | 15.29 |
| 200 | 21.43 | 15.06 |
| 300 | 21.72 | 15.02 |
| 400 | 21.77 | 15.08 |
| 500 | 21.59 | 14.98 |
| 600 | 21.41 | 14.95 |
| 700 | 21.57 | 14.89 |
| 800 | 21.31 | 14.98 |
| 900 | 21.36 | 14.91 |
| 1000 | 21.48 | 14.90 |

**Table 7.** Error rates in the process of forward variable selection for male cohort and female cohort based on the random survival forest algorithm.

| **Number of Variables** | **Error Rates(%)** | | **Number of Variables** | **Error Rates(%)** | |
| --- | --- | --- | --- | --- | --- |
|  | **Male Cohort** | **Female Cohort** |  | **Male Cohort** | **Female Cohort** |
| 1 | 35.78 | 27.86 | 26 | 20.76 | 14.32 |
| 2 | 28.80 | 21.78 | 27 | 20.80 | 14.38 |
| 3 | 25.58 | 18.92 | 28 | 20.81 | 14.45 |
| 4 | 21.60 | 14.78 | 29 | 20.80 | 14.45 |
| 5 | 21.08 | 14.48 | 30 | 20.94 | 14.41 |
| 6 | 20.85 | 14.34 | 31 | 20.91 | 14.43 |
| 7 | 20.85 | 14.33 | 32 | 20.84 | 14.50 |
| 8 | 20.99 | 14.27 | 33 | 20.96 | 14.47 |
| 9 | 20.93 | 14.34 | 34 | 20.93 | 14.50 |
| 10 | 20.92 | 14.20 | 35 | 21.05 | 14.49 |
| 11 | 20.87 | 14.22 | 36 | 21.10 | 14.51 |
| 12 | 20.85 | 14.23 | 37 | 21.09 | 14.62 |
| 13 | 20.81 | 14.22 | 38 | 21.24 | 14.64 |
| 14 | 20.68 | 14.18 | 39 | 21.18 | 14.61 |
| 15 | 20.84 | 14.19 | 40 | 21.17 | 14.66 |
| 16 | 20.75 | 14.21 | 41 | 21.19 | 14.67 |
| 17 | 20.69 | 14.26 | 42 | 21.25 | 14.66 |
| 18 | 20.72 | 14.21 | 43 | 21.36 | 14.71 |
| 19 | 20.67 | 14.28 | 44 | 21.37 | 14.82 |
| 20 | 20.65 | 14.32 | 45 | 21.28 | 14.77 |
| 21 | 20.72 | 14.23 | 46 | 21.33 | 14.83 |
| 22 | 20.64 | 14.23 | 47 | 21.26 | 14.81 |
| 23 | 20.75 | 14.30 | 48 | 21.38 | 14.84 |
| 24 | 20.72 | 14.36 | 49 | 21.42 | 14.96 |
| 25 | 20.81 | 14.37 |  |  |  |

**Table 8.** Summary of initial screening of MetS predictor variables in the male cohort.

| **Variables** | **The Univariate Cox Model (39)** | **The Lasso-Cox Model (34)** | **The RSF Algorithm (22)** |
| --- | --- | --- | --- |
| AGE | # | # | # |
| BMI | # | # | # |
| SBP | # | # | # |
| DBP | # | # | # |
| FPG | # | # | # |
| TG | # | # | # |
| HDLC | # | # | # |
| LDLC | # | # |  |
| DBIL |  |  |  |
| TBIL |  | # |  |
| ALB | # | # |  |
| AST | # |  |  |
| ALT | # | # |  |
| GGT | # |  |  |
| CH | # | # | # |
| CR |  |  |  |
| HB | # | # |  |
| LYM |  | # |  |
| NEU | # |  |  |
| RBC | # |  |  |
| WBC | # | # |  |
| PLT | # | # |  |
| TP | # | # | # |
| UA | # | # |  |
| UREA |  | # |  |
| ann_CumBMI | # |  | # |
| ann_CumSBP | # | # | # |
| ann_CumDBP | # | # | # |
| ann_CumFPG | # | # | # |
| ann_CumTG | # | # | # |
| ann_CumHDLC | # | # | # |
| ann_CumLDLC | # | # | # |
| ann_CumDBIL | # | # |  |
| ann_CumTBIL |  |  | # |
| ann_CumALB |  | # |  |
| ann_CumAST | # | # | # |
| ann_CumALT | # |  |  |
| ann_CumGGT | # | # | # |
| ann_CumCH | # | # | # |
| ann_CumCR |  |  |  |
| ann_CumHB | # |  |  |
| ann_CumLYM | # | # |  |
| ann_CumNEU | # |  |  |
| ann_CumRBC | # | # | # |
| ann_CumWBC | # |  |  |
| ann_CumPLT |  | # |  |
| ann_CumTP | # | # |  |
| ann_CumUA | # |  |  |
| ann_CumUREA |  |  | # |

# **represents the variable is selected significantly.**

**Table 9.** Summary of initial screening of MetS predictor variables in the female cohort.

| **Variables** | **The Univariate Cox Model (41)** | **The Lasso-Cox Model (39)** | **The RSF Algorithm (14)** |
| --- | --- | --- | --- |
| AGE | # | # | # |
| BMI | # | # |  |
| SBP | # | # | # |
| DBP | # |  |  |
| FPG | # | # | # |
| TG | # |  | # |
| HDLC | # | # | # |
| LDLC | # | # |  |
| DBIL | # |  |  |
| TBIL | # | # |  |
| ALB |  | # |  |
| AST |  | # |  |
| ALT | # | # |  |
| GGT | # | # |  |
| CH | # | # |  |
| CR |  |  |  |
| HB |  | # |  |
| LYM | # | # |  |
| NEU | # |  |  |
| RBC | # | # |  |
| WBC | # | # |  |
| PLT | # | # |  |
| TP | # | # |  |
| UA | # | # |  |
| UREA |  | # |  |
| ann_CumBMI | # |  |  |
| ann_CumSBP | # | # | # |
| ann_CumDBP | # | # | # |
| ann_CumFPG | # | # | # |
| ann_CumTG | # | # | # |
| ann_CumHDLC | # | # | # |
| ann_CumLDLC | # | # | # |
| ann_CumDBIL | # |  |  |
| ann_CumTBIL | # | # |  |
| ann_CumALB |  | # |  |
| ann_CumAST | # | # |  |
| ann_CumALT | # |  |  |
| ann_CumGGT | # |  | # |
| ann_CumCH | # | # | # |
| ann_CumCR |  | # |  |
| ann_CumHB | # | # |  |
| ann_CumLYM | # | # |  |
| ann_CumNEU | # | # |  |
| ann_CumRBC | # |  |  |
| ann_CumWBC | # | # |  |
| ann_CumPLT | # | # |  |
| ann_CumTP | # | # | # |
| ann_CumUA | # | # |  |
| ann_CumUREA |  | # |  |

# **represents the variable is selected significantly.**

**Table 10.** Construction parameters of the random survival forest model for the male cohort.

| **Construction of the male cohort random survival forest model** |
| --- |
| Sample size: 1527 |
| Number of deaths: 671 |
| Number of trees: 1000 |
| Forest terminal nodesize: 1 |
| Average no. of terminal nodes: 676.713 |
| No. of variables tried at each split: 12 |
| Total no. of variables: 15 |
| Resampling used to grow trees: swor |
| Resample size used to grow trees: 965 |
| Analysis: RSF |
| Family: surv |
| Splitting rule: logrank *random* |
| Number of random split points: 10 |
| (OOB) CRPS: 0.15859576 |
| (OOB) Requested performance error: 20.54% |

**Table 11.** Construction parameters of the random survival forest model for the female cohort.

| **Construction of the female cohort random survival forest model** |
| --- |
| Sample size: 3928 |
| Number of deaths: 1115 |
| Number of trees: 1000 |
| Forest terminal nodesize: 3 |
| Average no. of terminal nodes: 577.127 |
| No. of variables tried at each split: 9 |
| Total no. of variables: 9 |
| Resampling used to grow trees: swor |
| Resample size used to grow trees: 2482 |
| Analysis: RSF |
| Family: surv |
| Splitting rule: logrank *random* |
| Number of random split points: 10 |
| (OOB) CRPS: 0.11533527 |
| (OOB) Requested performance error: 14.51% |

**Table 12.** The comparison of potential predictor variables between MetS group and non-MetS group in the external validation male cohort.

| **Variable** | **MetS（n=94）** | | **Non-MetS（n=230）** | | ***Z*  *P*** | |
| --- | --- | --- | --- | --- | --- | --- |
|  | **M P_25_，P_75_** | | **M P_25_，P_75_** | |  |  |
| AGE | 32.00 | 28.00,38.00 | 34.00 | 29.00,42.00 | 8.44 | 0.097 |
| BMI | 24.21 | 22.68,26.32 | 22.95 | 20.65,24.80 | 10.97 | ＜0.001 |
| SBP | 129.00 | 120.25,135.00 | 123.00 | 117.00,132.00 | 10.35 | 0.011 |
| DBP | 80.00 | 75.00,82.00 | 77.00 | 72.00,82.75 | 10.10 | 0.046 |
| FPG | 4.98 | 4.67,5.26 | 4.85 | 4.56,5.10 | 10.43 | 0.006 |
| TG | 1.04 | 0.78,1.35 | 0.85 | 0.63,1.14 | 10.83 | ＜0.001 |
| HDLC | 1.12 | 1.04,1.28 | 1.28 | 1.14,1.48 | 6.69 | ＜0.001 |
| LDLC | 2.83 | 2.31,3.14 | 2.54 | 2.16,2.98 | 10.17 | 0.031 |
| DBIL | 3.85 | 2.83,5.28 | 4.65 | 3.50,5.68 | 7.75 | 0.001 |
| TBIL | 14.10 | 11.03,19.10 | 15.60 | 13.30,19.98 | 8.18 | 0.026 |
| ALB | 46.05 | 44.70,47.20 | 45.65 | 44.33,46.98 | 9.91 | 0.117 |
| AST | 21.00 | 19.00,25.00 | 21.00 | 18.00,24.00 | 9.70 | 0.265 |
| ALT | 24.00 | 18.00,30.75 | 20.00 | 15.00,26.00 | 10.77 | ＜0.001 |
| GGT | 18.00 | 14.00,25.75 | 18.00 | 14.00,24.00 | 9.55 | 0.437 |
| CH | 4.55 | 4.06,5.05 | 4.43 | 4.02,4.96 | 9.61 | 0.364 |
| CR | 74.00 | 70.00,80.75 | 77.00 | 71.00,82.75 | 8.46 | 0.104 |
| HB | 155.00 | 150.00,161.00 | 153.00 | 148.00,160.00 | 9.93 | 0.103 |
| LYM | 2.20 | 2.00,2.60 | 2.10 | 1.70,2.40 | 10.32 | 0.013 |
| NEU | 3.30 | 2.73,3.90 | 3.00 | 2.50,3.70 | 9.98 | 0.083 |
| RBC | 5.18 | 4.85,5.36 | 5.03 | 4.82,5.26 | 10.32 | 0.013 |
| WBC | 6.00 | 5.31,6.86 | 5.60 | 4.89,6.64 | 10.23 | 0.023 |
| PLT | 228.50 | 200.00,260.75 | 210.00 | 182.00,243.75 | 10.49 | 0.004 |
| TP | 72.65 | 71.00,74.60 | 71.85 | 69.40,74.10 | 10.19 | 0.027 |
| UA | 344.00 | 319.00,368.75 | 335.50 | 297.00,367.00 | 9.89 | 0.125 |
| UREA | 5.21 | 4.73,5.84 | 5.26 | 4.73,5.93 | 8.94 | 0.580 |
| ann_CumBMI | 24.75 | 22.98,26.34 | 23.14 | 20.93,24.91 | 11.20 | ＜0.001 |
| ann_CumSBP | 128.50 | 123.63,132.38 | 123.80 | 118.05,130.49 | 10.91 | ＜0.001 |
| ann_CumDBP | 79.56 | 75.53,82.80 | 76.63 | 72.24,81.00 | 10.77 | 0.001 |
| ann_CumFPG | 5.11 | 4.81,5.41 | 4.94 | 4.72,5.13 | 11.23 | ＜0.001 |
| ann_CumTG | 1.18 | 0.92,1.52 | 0.91 | 0.73,1.16 | 11.92 | ＜0.001 |
| ann_CumHDLC | 1.09 | 1.00,1.22 | 1.28 | 1.16,1.47 | 5.93 | ＜0.001 |
| ann_CumLDLC | 2.77 | 2.46,3.22 | 2.62 | 2.22,3.01 | 10.24 | 0.021 |
| ann_CumDBIL | 3.97 | 3.35,5.25 | 4.50 | 3.60,5.53 | 8.37 | 0.071 |
| ann_CumTBIL | 14.70 | 12.55,18.36 | 15.43 | 12.73,18.93 | 8.76 | 0.337 |
| ann_CumALB | 45.94 | 45.06,46.80 | 45.70 | 44.23,46.84 | 9.63 | 0.334 |
| ann_CumAST | 21.00 | 18.50,24.35 | 19.68 | 17.50,23.18 | 10.26 | 0.019 |
| ann_CumALT | 25.01 | 20.42,31.94 | 20.21 | 15.81,26.04 | 11.47 | ＜0.001 |
| ann_CumGGT | 20.05 | 15.26,26.73 | 17.76 | 14.00,24.88 | 10.03 | 0.065 |
| ann_CumCH | 4.57 | 4.11,5.13 | 4.56 | 4.18,4.97 | 9.34 | 0.742 |
| ann_CumCR | 75.24 | 69.31,81.81 | 75.00 | 69.52,81.39 | 9.38 | 0.682 |
| ann_CumHB | 154.50 | 149.31,161.48 | 151.50 | 144.05,158.50 | 10.61 | 0.002 |
| ann_CumLYM | 2.26 | 1.97,2.58 | 2.05 | 1.65,2.38 | 10.99 | ＜0.001 |
| ann_CumNEU | 3.13 | 2.70,3.69 | 2.80 | 2.24,3.45 | 10.65 | 0.001 |
| ann_CumRBC | 5.18 | 4.93,5.37 | 5.02 | 4.83,5.24 | 10.56 | 0.003 |
| ann_CumWBC | 6.11 | 5.30,6.98 | 5.64 | 4.97,6.54 | 10.21 | 0.025 |
| ann_CumPLT | 227.50 | 202.96,261.10 | 216.44 | 191.54,244.94 | 10.25 | 0.020 |
| ann_CumTP | 72.64 | 70.73,74.75 | 71.85 | 69.52,73.55 | 10.25 | 0.020 |
| ann_CumUA | 348.25 | 321.95,379.69 | 336.60 | 305.10,366.72 | 10.26 | 0.019 |
| ann_CumUREA | 5.16 | 4.71,5.73 | 5.28 | 4.74,5.87 | 8.94 | 0.571 |

**Table 13.** The comparison of potential predictor variables between MetS group and non-MetS group in the external validation female cohort.

| **Variable** | **MetS（n=402）** | | **Non-MetS（n=1213）** | | ***Z*   *P*** | |
| --- | --- | --- | --- | --- | --- | --- |
|  | **M P_25_，P_75_** | | **M P_25_，P_75_** | |  |  |
| AGE | 29.00 | 25.00,36.00 | 28.00 | 25.00,35.00 | 23.04 | 0.004 |
| BMI | 21.84 | 20.12,23.60 | 20.64 | 19.15,22.41 | 24.92 | ＜0.001 |
| SBP | 120.00 | 112.00,126.00 | 116.00 | 108.00,124.00 | 23.90 | ＜0.001 |
| DBP | 73.00 | 67.00,79.00 | 70.00 | 65.00,77.00 | 23.26 | 0.001 |
| FPG | 4.85 | 4.59,5.13 | 4.74 | 4.50,4.97 | 24.14 | ＜0.001 |
| TG | 0.77 | 0.57,0.99 | 0.64 | 0.53,0.82 | 24.70 | ＜0.001 |
| HDLC | 1.34 | 1.16,1.53 | 1.56 | 1.39,1.76 | 16.16 | ＜0.001 |
| LDLC | 2.41 | 2.06,2.76 | 2.31 | 1.95,2.64 | 23.35 | ＜0.001 |
| DBIL | 3.05 | 2.40,3.98 | 3.30 | 2.50,4.30 | 20.51 | 0.003 |
| TBIL | 12.30 | 9.90,15.38 | 13.00 | 10.30,16.50 | 20.68 | 0.009 |
| ALB | 44.85 | 43.30,46.40 | 44.80 | 43.40,46.20 | 21.86 | 0.894 |
| AST | 19.00 | 17.00,22.00 | 19.00 | 16.00,22.00 | 22.02 | 0.616 |
| ALT | 15.00 | 13.00,19.00 | 15.00 | 12.00,19.00 | 22.82 | 0.019 |
| GGT | 11.00 | 9.00,13.00 | 10.00 | 8.00,13.00 | 23.12 | 0.002 |
| CH | 4.31 | 3.84,4.70 | 4.38 | 3.98,4.77 | 20.73 | 0.013 |
| CR | 51.00 | 47.00,56.00 | 53.00 | 48.00,58.00 | 20.15 | ＜0.001 |
| HB | 131.00 | 125.00,136.00 | 131.00 | 125.00,136.00 | 21.93 | 0.771 |
| LYM | 2.00 | 1.70,2.30 | 1.90 | 1.60,2.30 | 22.33 | 0.223 |
| NEU | 3.20 | 2.60,4.00 | 3.00 | 2.30,3.70 | 23.48 | ＜0.001 |
| RBC | 4.38 | 4.20,4.60 | 4.40 | 4.18,4.59 | 21.89 | 0.842 |
| WBC | 5.83 | 4.90,6.65 | 5.43 | 4.67,6.43 | 23.42 | ＜0.001 |
| PLT | 239.00 | 210.00,270.75 | 236.00 | 205.00,270.00 | 22.01 | 0.625 |
| TP | 72.50 | 70.40,74.70 | 71.90 | 69.50,74.30 | 23.27 | 0.001 |
| UA | 244.00 | 215.25,279.75 | 250.00 | 219.00,279.00 | 21.51 | 0.508 |
| UREA | 4.49 | 3.90,5.24 | 4.49 | 3.82,5.29 | 22.02 | 0.619 |
| ann_CumBMI | 22.42 | 20.76,24.21 | 21.14 | 19.58,22.82 | 25.55 | ＜0.001 |
| ann_CumSBP | 122.74 | 116.44,127.95 | 116.83 | 111.42,122.01 | 26.55 | ＜0.001 |
| ann_CumDBP | 73.60 | 70.00,78.50 | 71.00 | 66.54,75.00 | 25.42 | ＜0.001 |
| ann_CumFPG | 5.03 | 4.80,5.28 | 4.87 | 4.69,5.06 | 25.54 | ＜0.001 |
| ann_CumTG | 0.88 | 0.69,1.15 | 0.74 | 0.60,0.90 | 25.89 | ＜0.001 |
| ann_CumHDLC | 1.32 | 1.17,1.46 | 1.57 | 1.42,1.75 | 14.64 | ＜0.001 |
| ann_CumLDLC | 2.51 | 2.20,2.81 | 2.40 | 2.05,2.73 | 23.29 | 0.001 |
| ann_CumDBIL | 3.20 | 2.60,3.96 | 3.53 | 2.83,4.41 | 19.46 | ＜0.001 |
| ann_CumTBIL | 12.35 | 10.10,14.68 | 13.27 | 10.97,15.87 | 19.73 | ＜0.001 |
| ann_CumALB | 44.54 | 43.47,45.55 | 44.80 | 43.76,45.85 | 20.43 | 0.002 |
| ann_CumAST | 18.00 | 15.79,20.99 | 17.49 | 15.50,20.00 | 23.03 | 0.005 |
| ann_CumALT | 15.74 | 13.00,20.77 | 14.57 | 12.07,18.00 | 23.76 | ＜0.001 |
| ann_CumGGT | 11.50 | 9.51,15.00 | 10.68 | 8.80,13.28 | 23.73 | ＜0.001 |
| ann_CumCH | 4.40 | 3.99,4.80 | 4.48 | 4.13,4.87 | 20.65 | 0.008 |
| ann_CumCR | 51.84 | 47.70,56.44 | 52.46 | 48.43,57.18 | 20.87 | 0.032 |
| ann_CumHB | 129.69 | 117.00,135.55 | 105.32 | 81.17,130.76 | 26.70 | ＜0.001 |
| ann_CumLYM | 2.05 | 1.72,2.39 | 1.94 | 1.63,2.27 | 23.06 | 0.004 |
| ann_CumNEU | 3.34 | 2.80,3.95 | 3.11 | 2.53,3.77 | 23.50 | ＜0.001 |
| ann_CumRBC | 4.40 | 4.24,4.61 | 4.40 | 4.23,4.58 | 22.21 | 0.349 |
| ann_CumWBC | 5.90 | 5.30,6.77 | 5.68 | 4.90,6.45 | 23.71 | ＜0.001 |
| ann_CumPLT | 247.24 | 216.63,280.50 | 242.39 | 212.59,276.28 | 22.34 | 0.212 |
| ann_CumTP | 71.87 | 70.12,74.01 | 71.65 | 69.89,73.61 | 22.62 | 0.059 |
| ann_CumUA | 249.50 | 225.63,283.86 | 253.50 | 228.89,279.76 | 21.73 | 0.872 |
| ann_CumUREA | 4.58 | 4.01,5.11 | 4.48 | 3.95,5.11 | 22.13 | 0.446 |
